# SNP hot-spots in the clam parasite QPX

**DOI:** 10.1186/s12864-018-4866-8

**Published:** 2018-06-20

**Authors:** Sleiman Bassim, Bassem Allam

**Affiliations:** School of Marine and Atmospheric Sciences, Stony Brook University, NY, 11794-5000 USA

**Keywords:** Pathogen, Mutations, Virulence, Adaptation

## Abstract

**Background:**

Quahog Parasite Unknown (QPX) is an opportunistic protistan pathogen of the clam *Mercenaria mercenaria*. Infections with QPX have caused significant economic losses in the Northeastern United States. Previous research demonstrated a geographic gradient for disease prevalence and intensity, but little information is available on the genetic diversity of the parasite throughout its distribution range. Also, QPX virulence factors are not well understood. This study addresses the occurrence of QPX genetic variants with a particular focus on functions involved in virulence and adaptation to environmental conditions.

**Results:**

Analyses were performed using transcriptome-wide single-nucleotide polymorphism (SNP) of four QPX isolates cultured from infected clams collected from disparate locations along the Northeastern United States. For contig assembly and mapping, two different genome builds and four transcriptomes of the parasite were examined. Genomic variants appeared at a differential rate relative to sequenced transcripts at 20.18 and 22.55% occurrence under 1000 base pairs upstream and downstream protein domains respectively and at 57.26% rate in protein domain coding sequences. QPX strains shared 30.50% of the mutations and exhibited a preferential nucleotide substitution towards thymine. Sequence identity suggested relatedness between different QPX strains, with the parasite being possibly introduced to Virginia from the Massachusetts region during clam trading, while QPX could have been naturally present in New York. Diversity in virulence, temperature, and salinity domains suggested a common variability between strains, but with a preferential higher variation in local adaptation genes. This could explain differences in disease prevalence noted in different regions. Overall, the results supported views that this opportunistic parasite might be able to adapt to varying environmental conditions.

**Conclusion:**

Relatedness and mutations between the four QPX strains suggested that variability in environmental-related functions favors parasite survival, potentially promoting resilience against stressful conditions. These findings are in agreement with the widespread presence of QPX in the environment. Although QPX levels are enzootic in most areas, an increase in disease outbreaks were often associated with seasonal changes in environmental conditions. A selection mediated by the parasitic life of QPX remains possible, but the effect of the environment on the biology of the parasite appears more obvious.

**Electronic supplementary material:**

The online version of this article (10.1186/s12864-018-4866-8) contains supplementary material, which is available to authorized users.

## Background

Quahog Parasite Unknown (QPX) is an opportunistic protistan pathogen of the commercially harvested hard clam *Mercenaria mercenaria*. Infections with this pathogen have caused significant economic losses in the Northeastern United States and Maritime Canada. Previous research demonstrated strong effect of host genetic background on disease outcome, which made a population either resistant to infection or susceptible with high mortality rates [1, 2]. Environmental conditions were also shown to strongly regulate disease development both in aquacultured and wild clams [3–7]. In this framework, environmental factors such as temperature and salinity were shown to affect parasite growth and virulence [6, 8] as well as clam immune performances [4, 6]. Accounting for these abiotic conditions uncovers associations between evolutionary traits during host-pathogen interaction and adaptation mechanisms that increases the fitness of an organism in a particular environment.

Like other pathogens, QPX population structure and spread are supposedly changing based on the physiological responses of its host [9, 10]. Adjustments associated with the adaptive behavior of a pathogen and its performance are often influenced by single-nucleotide polymorphisms (SNPs) that contribute to alteration of gene expression, protein synthesis, and activity. Prior investigations have shown that temperature had a direct effect on the expression of growth and virulence genes responsible for adaptation mechanisms and pathogenicity, which appeared to also contain an enriched number of SNPs compared to maintenance and metabolism genes [11]. However, no analyses have focused on understanding the polymorphism of QPX genome and its relationship to the parasite distribution in North America. Furthermore, no specific and influential genomic mutations were identified for this pathogen, even though previous observations showed differential pathogenicity among various QPX strains [1, 12, 13]. Therefore, characterizing the polymorphism of QPX strains based on conserved protein domains was essential for evaluating the genetic basis of geographic differences within the species.

The first aim in this study was to create a preliminary model based on SNP predictions that would associate a genetic divergence to the various QPX strains. Comparison was made using transcriptomes of four QPX strains that derived from geographically different locations in three states. Differential distribution of SNPs and a preferential substitution of nucleotides were observed between strains. QPX transcripts were then annotated and genetic variants were investigated inside and outside functional domains. Higher number of variants were localized outside commonly known virulence protein domains while many others were found inside protein domains related to adaptation to environmental conditions. The results were analyzed with a focus on the identification of possible trade-offs between QPX virulence and adaptation to local environmental conditions.

## Methods

Two guidelines are available on GitHub [14]. They detail all phases of the protocol to test and analyze the RNA-seq raw reads, the R code and figures (Additional File 2), as well as steps for sequence annotation and variant calling [15]. Listing of all packages, parameters used, and alternatives to these tools is given in the supported online texts. The bioinformatic pipeline, data mining, and R statistical inferences were collectively performed on the high performance computing server (Bridges) of the Extreme Science and Engineering Discovery Environment (XSEDE, [16]).

### Parasite samples

Four QPX strains were isolated from infected clams collected from four different locations in the United States. Two strains originated from clams collected from two polymorphic sites in New York, labeled as MMETSP0098 (8 BC7 from Raritan Bay; Lat 40.498156, Lon − 74.165342) and MMETSP1433 (3408D from Peconic estuary; Lat 40.908312, Lon − 72.591262), one strain from Massachusetts labeled as MMETSP0099 (ATCC 50749), and a final strain from Virginia labeled as MMETSP0100 (374A1; Lat 37.231442, Lon − 75.996817). Isolated strains were cultured in vitro and vegetative stages of QPX (thalli) were retrieved at the exponential stage of growth as previously described [13]. QPX strains sequencing was part of the Marine Microbial Eukaryote Transcriptome Sequencing Project (MMETSP), which accomplished sequencing of 678 marine samples including 305 microbial species and 409 unique strains [17]. RNA from the four environmental isolates was sequenced at the National Center for Genome Resources (NCGR). Protocols and guidelines are available for RNA extraction and sequencing in [17]. Briefly, we prepared a library for each QPX strain with the Illumina HiSeq 2000 platform, in paired-ends at 100 bp for over 2 Gbp sequences.

### RNA-seq assembly and annotation

RNA-seq raw reads are found at the National Center for Biotechnology Information (NCBI) under the BioProject accession number PRJNA248394 at http://www.ncbi.nlm.nih.gov/bioproject/248394. We used the appropriate sets of Illumina TrueSeq default adapters for paired-ends for quality testing, also necessary for trimming low quality nucleotides. We checked the reads for genomic quality scores before and after PHRED 33 quality-trimming with trimmomatic v0.33 [18] and mapped filtered reads with Burrows-Wheeler aligner (BWA-MEM) v0712.r1039 [19] to six different QPX references that included four transcriptomes (described above) and two draft genomes. We used Trinity v2.0.6 [20] to assemble the QPX transcriptomes (all scripts and parameters are available in the supporting online text). MMETSP assemblies were constructed with the BPA v2.1.0 assembly pipeline and the detailed tutorial is available on GitHub at [21] or the published work of [17]. We analyzed the phylogeny of the assembled contigs using Phylosift v1.0.1 [22] that also integrate pplacer v1.1 [23], which assumes a minimum likelihood-based phylogenetic placement of sequences. Phylosift’s core marker set helped align contigs to find the nearest neighbor of the four QPX strains and to categorize the nearest reference among the strains. Mauve v2.4.0 [24] was then used to order the assemblies. Finally, we accomplished a phylogenetic placement by homologous sequence comparison. The hierarchical clustering between samples was calculated with Kantorovich-Rubenstein distances [25]. We used HMMER v3.1b1 [26] to align functional protein domains from the Pfam database v28 [27] to the assembled contigs of the QPX strains, by translating them in three frames into amino acid sequences with Transeq from Emboss v6.5.7. We selected virulence, temperature, and salinity domains by text mining of the Pfam database. Interpro accession numbers and Gene Ontology (GO) terms were generated on the identified and retained orthologs.

### Genetic variants calling

The reference genome used (QPX v1 Fig. 2) was from [11] and helped for genetic variant calling and mapping analyzes. The QPX strain used for the reference genome sequencing originated from Massachusetts, United States. We called SNPs with the Genomic Analysis Toolkit (GATK) v3.4–46 [28], and used SAMtools v1.2 [29], VCFtools v0.1.13 [30], and BCFtools v1.2 [31] for variant processing. First, we called indels then realigned reads around identified indels with GATK. This step reduced bias by correcting the shift in read alignment if a variant was wrongly called at one position. GATK was also used to recalibrate probabilities of the variant calling confidence scores, necessary to adjusts for calling biases. We used a hard filtering phase to select variants for each of the four QPX strains by discarding low quality calls using a confidence score (a = 95%) and the depth of coverage as metrics (qual = 2.0; fs = 60.0; mq = 40.0; sor = 3.0). This step was optional but increased the confidence of variant calling since we lack a reliable annotated genome. We used SNPs with an estimated Phred quality score of Q33 or greater for diversity analyses. We also compared mapped reads to different references for quality control and to select the most compatible parameters (N50, depth of coverage, and overall mean contig length) and used Samtools v1.2, Picard-tools v1.27, and HTSeq v0.6.1 [32] for quality scoring, sorting to reference, and removing duplicate sequencing products. Contigs for every QPX assembly were then aligned to the reference genome with BLAT [33]. This located genetic variants in each of the QPX assembled transcriptome. We set the minimum sequence identity at 90% between genome and transcriptome contigs alignment. Contigs were frequently found multiple times in the genome at different alignment lengths. Therefore, the mean size of the QPX contigs was critical to find the correct number of mapped sequences and setting a maximum alignment coverage to eliminate redundancy, since the PCA analysis had shown (Fig. 3) that a longer alignment was significant to distinguish between strains. To accomplish one genomic alignment per contig and thus avoid genomic redundancy, the alignment length had to be at least equal to the half of the mean of the contigs sizes for each of Pfam selected categories (Additional File 2).

## Results and discussion

### Relatedness and classification of QPX references

**Fig. 1:**
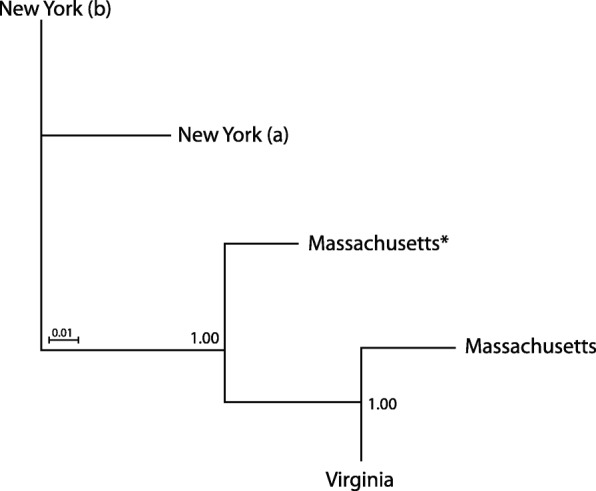
Phylogenetic placement by homologous sequences of four QPX strains. Hierarchical clustering between samples after aligning amino acid sequences between QPX assembled contigs to phylogenetic markers (available in the supporting online text). New York (a) and (b) are two isolated QPX strains originated from disparate locations in New York. (*) represents the reference genome of QPX (v1) used for base-calling from Fig. 2. The scores represent the level of certainty at least two strains show when placed near one another

Four genetically distinct QPX strains were isolated from three different states. Two isolates came from New York (NY), one from Massachusetts (MA), and another from Virginia (VA). RNA sequencing of these strains was part of an international project with the aim to sequence a wide number of eukaryotic marine microbes [17]. Around 100 markers were selected to search and compare homologs between the four QPX strains and the reference genome (list of markers is available in the supporting online text). A phylogenetic placement was generated between these strains with pplacer based on peptide alignment with HMMER (Fig. 1). Distances among samples represented a hierarchical clustering of the weighted likelihoods (uncertainty placements) of the aligned homologues. A placement score of 1, on a scale from 0 (uncertain) to 1 (certain) as a penalized distance, helped classify which set of contigs were significant to be used in further analysis.

**Fig. 2:**
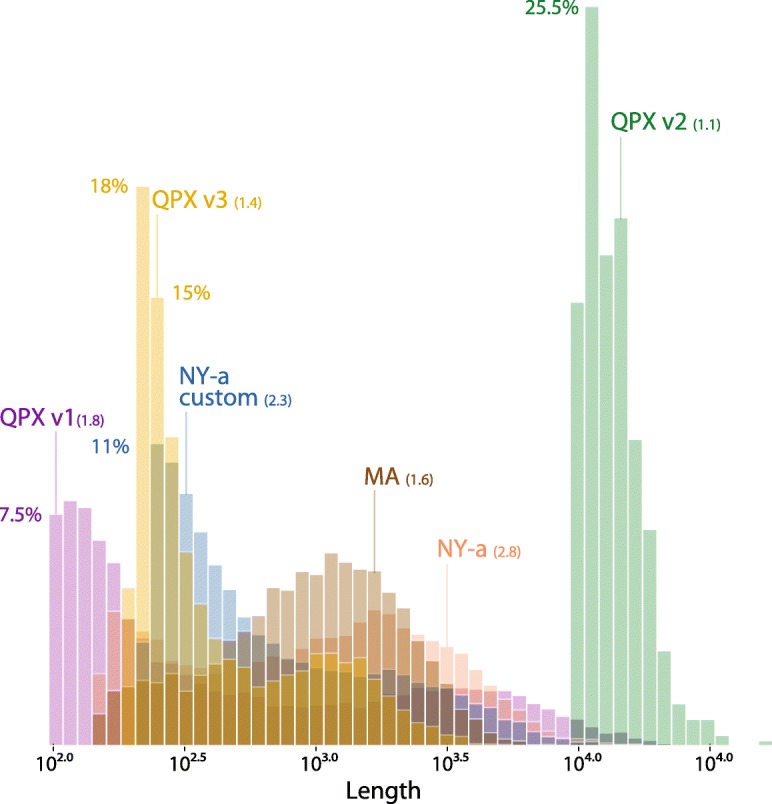
Contig length of references used for variant calling. The distribution of length for each genome and transcriptome assembly tested for variant calling. Two QPX genomes builds (v1, v2) and four QPX transcriptomes are compared. N50 scores are given between parentheses and the y-axis depicts the percentage of the total assembly counts

Two genome builds were tested to estimate biases in QPX transcriptome contig assemblies and compare parameters used for each build. Both builds and one transcriptome were specific to the MA QPX strain [11]. Two other transcriptomes were specific to a NY QPX strain, both assembled with different thresholds (supporting online text). The MA genome (QPX v1) was selected for all subsequent genetic variant calling [11]. This reference contained approximately 22,000 contigs, covering 34.66 Mbp (Additional File 1 Figure S1-A). The distribution of contigs length of the QPX reference genomes and all distinct transcriptomes is shown in Fig. 2. The quality and comparison tests were evaluated for all the transcriptomes (contigs counts and length, mean contig length, N50, and L50).

A fast annotation protocol [34] was used for quality checks that delivered descriptive analysis on the four QPX transcriptomes and the selected reference genome. A comprehensive analysis of functional sequences for all QPX assembled transcripts helped identify top processes, molecular mechanisms specific to conserved proteins, and overall sequence similarities important for phylogenetic relatedness (Additional file 1 Fig. S2). Data showed homogeneity of read length and content across the sequenced QPX samples. Additionally, the phylogenetic classification of sequencing reads helped assess the efficient isolation of QPX transcripts.

**Fig. 3:**
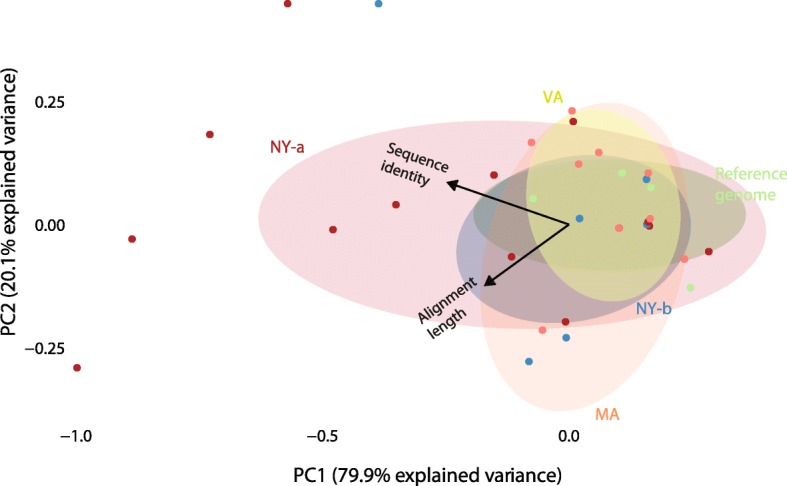
Differences among four QPX strains and with the reference genome. The principal component analysis is based on 100 annotated functional sequences that are selected after MG-RAST quality-control checks of the annotation process and sequence similarity search on 5 QPX libraries that include four QPX strains and the reference genome. Feature selection is done with an identity score (maximum of 1 mismatch) for annotating the QPX contigs against protein domains, an alignment length score (similarity ≥ 80%) that represent the coverage length and similarities between estimated alignments, an e-value score (10^-10^) for predicted functional similarities, and the number of times an annotated contigs is identified. The arrows are eigenvectors, they represent linear trends in the data

A principal component analysis showed relatedness between the selected QPX genome and the distinct transcriptome assemblies on the basis of sequence closeness and transcript annotation (Fig. 3). The top 100 annotated QPX transcripts were examined after ranking the contigs based on the length of coverage of sequence alignment (minimum of 200 base pairs) and the abundance of annotated sequences at an e-value of 10^-10^. The analysis of sequence identity suggested a relatedness between QPX strains based on a functional classification of their annotated transcripts [35]. It would be possible to consider that MA and VA strains were overall closer to each others, than to the NY strains. This would suggest that QPX was probably introduced to Virginia from the Massachusetts region (or, less likely given disease history in both states) during clam trading or other exchanges, while QPX could have been naturally present in New York. In addition, the total number of transcripts was not taken into account, because examinations were made preferably using length and transcript functional coverage. This helped test sequence compatibility between QPX libraries and the reference genome, which were relevant to map the density of SNPs and their 5′ start 3′ end intergenic/exonic location [36].

### QPX genetic variants and their potential effect on disease transmission

Presently, QPX had no exhaustive list of annotated SNPs, hence the reliance on a longer approach for SNP calling. The selected QPX (v1) reference genome (Fig. 2) was used for genetic variant calling following the Genomic Analysis Toolkit (GATK) standard procedures. Although one reference genome was used in all subsequent analyses, all variants were tested through several iterations and were mapped to the four available QPX transcriptomes (supporting online text). Initially, preliminary variant calling runs were performed under stringent thresholds and three rounds of filtering to remove and reduce recall [37]. This was important because for each QPX strain and during each iteration, a different list of variants was selected to test precision error estimation of the procedure (data not shown). These variants subsets were not final, but were generally used to train the GATK algorithm to increase the confidence score of each base called on a transcriptome-wide scale. GATK parameters included mapping quality raw and posterior positional probabilities for each base called, which helped estimate each variant quality (uncertainty score) and likelihood [38]. Using scores from depth of coverage data, the positional quality of bases called delivered a confidence estimation for each mapped read, which was used for error correction and recalling of relative variants [39]. The final list was then used to recalibrate the quality scores of reads in each transcriptome, thus refining the mapping procedure [40]. The previous step was repeated twice using loosened mapping filters on the scores calibrated transcriptome to increase variant identification and recall. Theoretically, the base calling likelihood was recalibrated twice, then variants were identified at three different iterations. This multi-step approach, based on iterative variant calling, recalibration, and correction of estimated base calling, delivered a higher confidence list of variants.

To investigate sequence variation between QPX strains, SNP densities were estimated at a resolution of 1000 base pairs from assembled transcripts. Results showed a close pairwise genetic diversity between Virginia (VA) and Massachusetts (MA) strains with 0.45 and 0.49 SNPs per 1 Kb respectively and between both New York (NY) isolates (a and b) with 0.67 and 0.70 SNPs per 1 Kb respectively. Similar estimated densities were presumably observed in replicated data when evaluating the effect of temperature on gene response of the MA QPX isolate [11]. This transcriptome-wide diversity reflected geographical divergence between NY isolates and both MA or VA strains. Previous studies showed similarities in disease features among different geographic regions [41–44]. However, major differences in disease prevalence also existed between MA and VA, while QPX isolates seemed rather genetically close [1]. This was not surprising since disease development also depended on the effect of environmental factors. These conditions, which were very different between MA and VA, were known to impact clam resistance as well as parasite fitness and host-parasite interactions. Based on the observed high diversity scores, NY strains might indeed be more virulent than MA and VA strains, as suggested earlier by Dahl et al., [1].

Additionally, prior work showed that environmental factors affected host fitness and immune performances leading to enhanced susceptibility or, alternatively, resistance to QPX [3, 4, 6, 45]. For example, disease prevalence and intensity were markedly higher in clams exposed to low temperatures (13 °C) as compared to animals held at milder temperatures (21 °C and 27 °C) [46]. Interestingly, QPX exhibited adaptation to a wide range of salinity conditions that spanned from 20 to 40 ppt [4, 47]. It was possible that the higher genetic diversity in the NY strains might confer QPX a wider adaptability to varying environmental conditions, particularly for temperature and salinity (detailed below). These assumptions will be verified further in this report by comparing the rates of variants in both virulence and local adaptation genes in each strain.

**Fig. 4:**
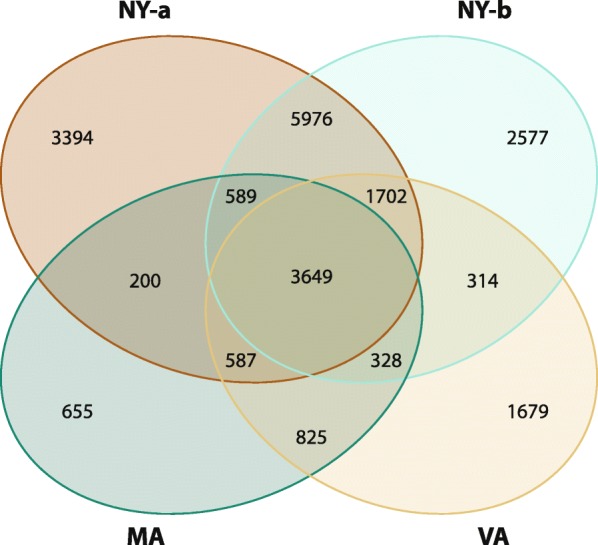
Number of shared and unique SNPs between four QPX strains. A count of SNPs that were identified either in one QPX strain, between 2 strains, common in 3 strains or shared among all sequenced strains

Genetic exchange was examined between QPX strains by evaluating the distribution of SNP segmentation patterns in the reference genome. Regression of the position of each SNP on its quality score provided a uniform concentration of SNPs in the first 10 Kb for the QPX transcripts (Additional file 2). The depth of coverage was over 50 along the first 10 Kb and showed an occurrence of a minimum of 11.41% of the SNPs. Indeed, SNP count was relative to quality thresholds, which can be lowered if necessary, but at a 50 cutoff false positives were efficiently reduced and loss-of-sensitivity was negatively correlated to bases called in longer contigs [38, 48]. Geographical and evolutionary distances influence SNP rates in orthologous genes [49], but despite the local proximity of isolates, NY QPX strains showed in this report high SNP rates. Nucleotide substitution comparison in orthologous sequences between the four QPX strains suggested a presence of different selection pressures, probably a consequence of environmental factors or divergence in the evolutionary process. Figure 4 illustrates the number of unique and common SNPs between each QPX strain. The supporting online text also includes indel counts (Additional file 1 Figure S3). The highest SNP shared rate was equal to 5976 in 1422 QPX orthologs and was specific to the NY isolates, which despite the low number of parasite samples, indicated a potential geographical relatedness. All four strains shared 3649 SNPs in 1824 QPX orthologs. Both NY isolates and the VA strain exhibited the highest count of unique SNPs with 3394, 2577, and 1679 respectively and the number of SNPs common among them was 1702 SNPs, which was found in 567 QPX orthologs. This suggested the potential existence of an acting positive selection pressure on the genes of each strain and the occurrence of potentially distinct selective constraints. Furthermore, the MA strain showed lower SNP count presumably because the reference genome was geographically close.

**Fig. 5:**
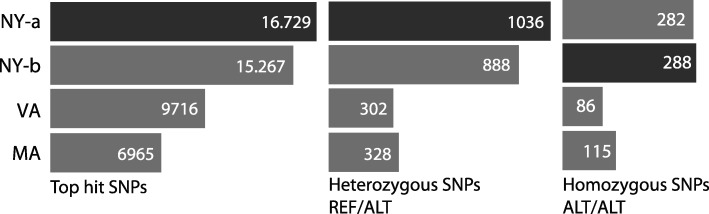
Number and nature of called SNPs between QPX strains. A count of SNPs called that figure in a homozygous and heterozygous distribution between the reference and the four QPX strains

In addition to the polymorphism between strains, nucleotide polymorphism specific to each strain was also examined. The homozygous and heterozygous patterns in SNP calls were identified, but first all alleles were excluded with a maximum confidence probability of 90% outside 10 Kb of the reference genome by normalizing SNPs with phred scaled likelihoods (L). Phred likelihoods (PL) helped adjust accuracy of scores for homozygosity and heterozygosity using probability estimates from the following eq. $\mathrm{P}\kern.1em \left(\mathrm{L}|\mathrm{AA}\right)={10}^{-{\mathrm{P}}_{\mathrm{AA}}/10}$ [38]. Figure 5 showed the number of variants called for each QPX strain and the count of homozygous and heterozygous mutations identified. Both NY isolates were distinguished with a high SNP count in both genotypes, except that NY-b strain showed more homozygous SNPs. This was intriguing because NY-b had a tendency in showing lower counts (reads, contigs, SNPs) than the first strain. Indeed, deeper QPX sampling for more populations (sample size and demographic origins) was required to explain these diversity patterns with better precision, hence increasing the resolution of relative allele frequencies for each strain. Synonymous and non-synonymous SNPs were not examined because an annotated genome of QPX is not yet available, which would increase precision of QPX associations to environmental factors or functional classification. For this reason a work on an annotation pipeline for the QPX genome would be essential for further analysis, which will help future characterization of any purifying pressures of potential QPX orthologs.

### Distribution of QPX SNPs in virulence and adaptation transcripts

**Fig. 6:**
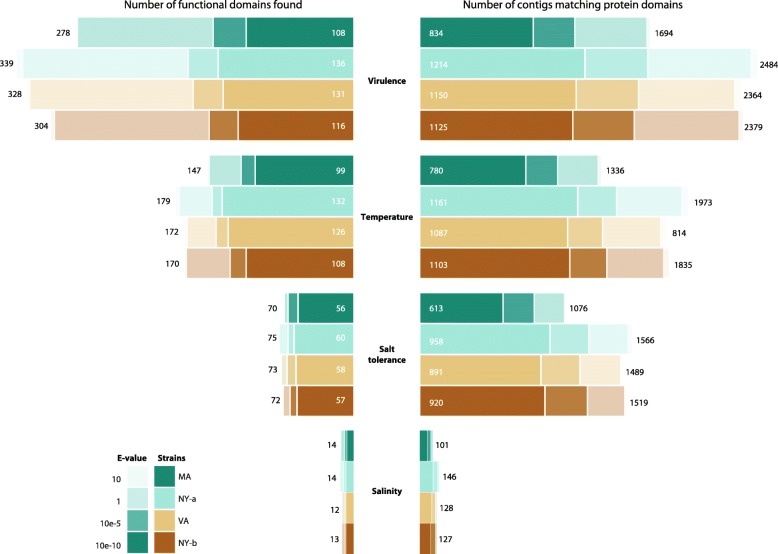
Number of Pfam functional domains or aligned contigs for four QPX strains. Pfam is a public database that includes functional protein domains found in many species. Alignment between QPX contigs and Pfam domains is done with HMMER for each sequenced strain. Each color represents a strain. Each contrast for each color represents a different e-value. Virulence, temperature, salt tolerance, and salinity are the selected Pfam domains used for QPX annotation. On the left is the unique number of domains identified from the alignment. For example, if 2 different contigs are aligned to one Pfam domain 1 is added. On the right is the estimated number of QPX contigs with protein domains. For example if 2 different contigs are aligned to one Pfam domain 2 are added

To examine the possible reasons for genetic differences amongst strains, we annotated QPX contigs with Pfam protein domains. All functions related to infectivity and local adaptation were selected and were loosely referred to as virulence, temperature, and salinity proteins. These conserved protein families were either directly or remotely implicated in virulence and adaptation in the Pfam database (v28). A search of the Pfam database resulted at an e-value score of 10^-10^ different QPX transcripts with 655 virulence, 251 temperature, 79 salt tolerance, or 22 salinity protein related domains. Alignment probability scores were evaluated with Hidden Markov Models implemented in HMMER, which helped select most significant domains and least redundant annotated contigs (Fig. 6). Functional annotation of contigs was on the basis of e-value scores, percentage of identity between contigs and protein domains, and coverage score that implied conservancy of matching domains.

**Fig. 7:**
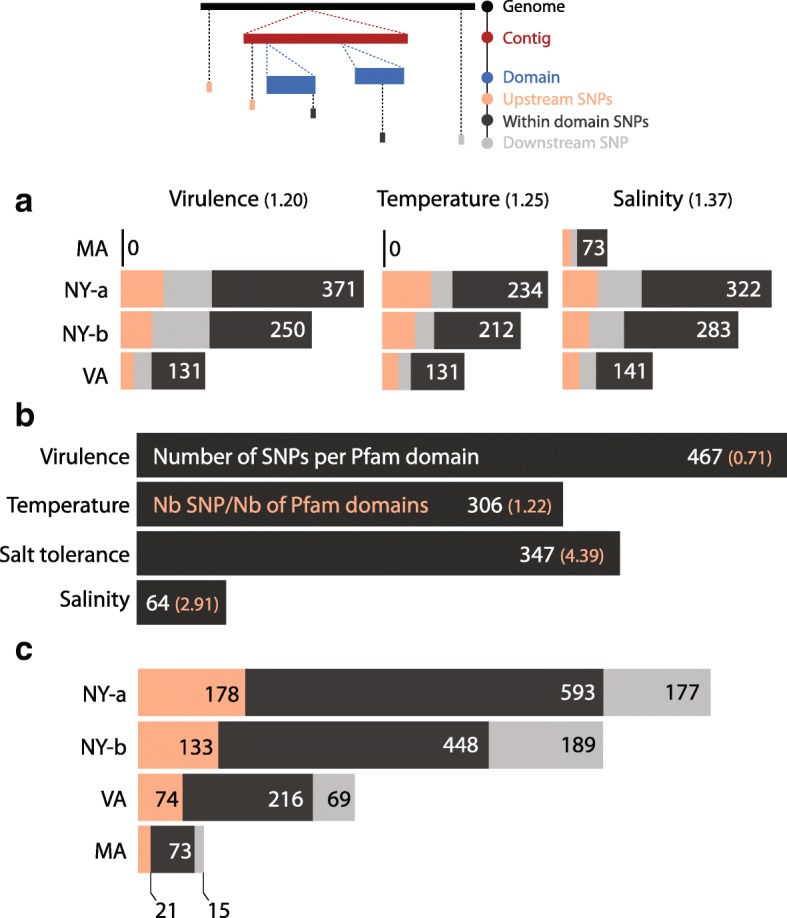
SNP counts per Pfam domain and per QPX strain. The schematic graph illustrate the presence (in color) of SNPs inside (black) and outside (orange and gray) targeted QPX protein domains. QPX libraries used are those of New York (NY), Massachusetts (MA), and Virginia (VA). Contig annotation at e-value ≤ 10^-10^ and SNP calling after two recalibrations. **a**- Number of SNPs within (black) and outside (orange and gray) Pfam domain. **b**- Standardized number of SNPs for each Pfam category (white). The occurrence score between parentheses is the number of SNPs within Pfam domains normalized by the sum of the total size of QPX contigs that align to each of virulence, temperature, salinity domains. The number of Pfam domains is different between virulence and adaptation so we divided the SNP count by the number of domains retrieved from Pfam (orange). **c**- Number of SNPs for each QPX strain

**Fig. 8:**
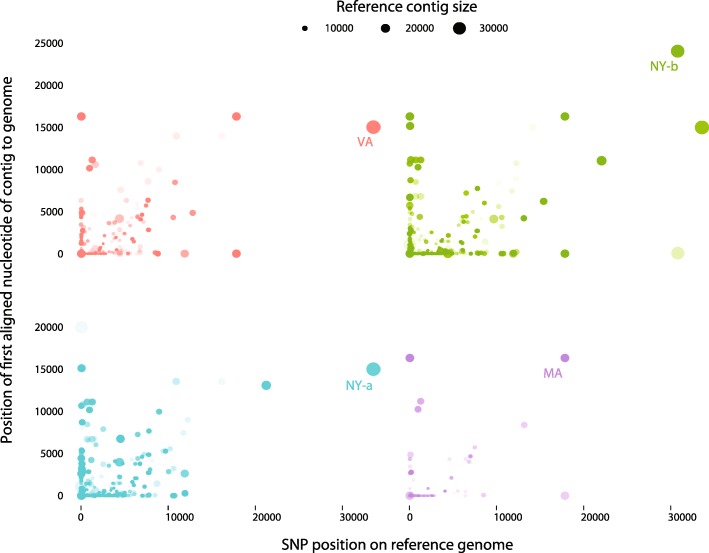
Distribution of SNPs inside and outside contigs of QPX related to virulence and adaptation. Location of SNPs to the protein domains was inferred after alignment of the contigs to the reference genome. The x-axis shows the position of SNPs on the reference genome. The y-axis shows the genomic position from aligning the contigs with predicted protein domains to the reference genome. The size of the dots represent the sequence size of the reference genome. VA: Virginia strain in red, NY-a,b: New York strains a and b in green and blue respectively, and MA: Massachusetts strain in purple

The density of QPX SNPs was characterized inside and outside the annotated domains for each strain. After predicting QPX protein domains and calling variants with the selected reference, contigs were aligned to the genome and SNP location was estimated in the domain-coding sequences. Results showed that 57.26% of SNPs identified in transcripts linked to virulence, temperature, and salinity were in protein domains, while a total of 42.73% SNPs resided outside of these domains, grouped between 20.18% upstream and 22.55% downstream contigs (1 Kb; Fig. 7). Figure 8 summarizes the density and position of SNPs distributed between regions coding for the targeted functional transcripts (temperature, salinity, and virulence-related) and those located outside of these regions. Theoretically, a variant was inside a targeted coding sequence if its position in the genome was higher than the position of the first aligned nucleotide between the genome and the contig. For example, if the x-axis value of a variant in Fig. 8 was higher than its y-axis value then the variant was supposedly inside a coding sequence. However, if the x-axis value of a variant was lower than its y-axis value then the variant was upstream, before the 5′ start of the targeted QPX contig. Indeed, the length of the alignment coverage was crucial in determining the placement of variants. Therefore, a downstream SNP was outside of the 3′ end of the QPX contig, only when the x-axis position of the SNP was higher than the position of the last aligned nucleotide between the genome and the contig (data not shown).

Previously, it was found that the bases called in all four QPX strains were homogeneously distributed along the genome at a low rate of segmentation patterns (supporting online text). Therefore, SNP distribution was examined in QPX annotated transcripts to compare their occurrence between virulence, temperature, and salinity functions. First, a difference in distribution of SNPs among QPX orthologs supposedly contrasted with a difference in parasite biology and possibly virulence. For example, it was suggested that depending on the environmental conditions, QPX strains produced various levels of virulence-related proteins [50]. Additionally, since protein synthesis is dependent also on gene regulation, genomic variants in coding sequences and regulatory DNA regions could significantly affect microbial fitness. Therefore, finding genetic variability between isolates potentially indicates physiological differences between QPX strains.

Second, a selective genetic diversity was assumed to show correlation with different evolutionary selection pressures. Indeed, the tug of war between hosts and parasites often lead to a continuous evolution of virulence and resistance processes. As such, selection pressures that alter host resistance frequently include changes in environmental conditions [51]. Therefore, when considering long term exposure, a parasite transmission could be reduced if the only available host belongs to a resistant population [52], but in high density susceptible populations the pathogen transmission was reported to increase [53], particularly in the case of directly transmitted pathogens [54]. For example, a parasite was efficiently transmitted when inbreeding susceptible host populations were dominant (e.g., [55]). It should be noted however that QPX is an opportunistic parasite and its presence in an environment can be independent from clam infection. Additionally, since clams are sessile organisms, they may display a relatively restricted gene flow, further promoting disease spread among susceptible populations [56–58]. However, a localized population collapse of clams due to severe QPX outbreaks could also reduce pathogen transmission. In sum, these observations could presumably support the existence of a trade-off between pathogen transmission and virulence of a parasite that causes severe mortality events [59]. For these reasons, we further focused our investigation on contrasting SNP distribution between virulence and adaptation transcripts.

Virulence and adaptation transcripts were estimated to have a high mutation rate and belonged to 6.7% of the QPX transcriptome, which included presumably around 15,000 transcripts [8, 11]. Therefore, among all QPX strains, the functional transcripts for virulence, temperature, and salinity held 752, 577, and 819 SNPs respectively (Fig. 7-A). If one SNP was called every 2 Kb and the mean size of all contigs was 3 Kb (supporting online text), the virulence and adaptation transcripts with 2148 identified SNPs inside coding regions would have spanned a total length of 4300 Kb and thus accounted for a minimum of 700 QPX contigs. However, the total number of contigs found that contained these SNPs was actually 605 contigs specific to 139 different virulence and adaptation functions. Indeed, the size of contigs was relevant in this analysis and Fig. 8 shows that contig sizes were uniform between strains and SNP positions within strains. Contrary to what was expected, these SNPs were enriched in fewer contigs, therefore the genetic diversity of the virulence and adaptation transcripts was presumably higher than the rest of the genome.

In addition, the number of virulence and adaptation (temperature, salinity, and salt tolerance) domains used to annotate QPX contigs was not identical, so by normalizing the SNP counts with the number of Pfam domains, a frequency score was estimated that distinguished which category was less genetically diverse. Among the four QPX strains, only 108–136 domains were found among the 655 virulence domains retrieved from Pfam (Fig. 6). They matched 834–1214 unique contigs (e-value 10^-10^). Therefore, the virulence transcripts showed a low occurrence score of 0.71 (Fig. 7-B). This suggested that virulence genes in these four QPX strains might also be less diverse than those associated with local adaptation. Moreover, the most genetically diverse annotated transcripts included temperature domains with an occurrence score of 1.22, salinity at 2.91, and salt tolerance with the highest divergence occurrence score of 4.39, possibly allowing the parasite to prosper under varying salinity conditions. A range of 613–958 unique QPX transcripts contained 79 salt tolerance domains from Pfam, among which 56–60 domains (e-value 10^-10^) existed in all four QPX strains (Fig. 6). Inferring a normalized SNP density of the 79 domains estimated the presence of 347 SNPs, a higher score than of temperature with 306 SNPs for 251 domains.

Parasite strains presumably differed in their response even if they shared the same molecular mechanisms. Therefore the potential of each strain was investigated to evolve a different level of pathogenicity or was preferentially promoting local adaptation through an increase in genetic diversity of temperature- and salinity-related transcripts (Fig. 7-c). The percentage of SNPs found for both NY isolates in all virulence and local adaptation transcripts was 5.7 and 5.0% (of the total SNP count of each strain), for the VA strain it was 3.7%, and for MA strain it was 1.6%. Of course, the low score in the MA isolate can be attributed to the relatedness between the strain and the reference genome used for base calling. However, the SNPs called from the MA isolate only this time with different references (NY origin) also inferred a low SNP score (Additional file 1 Figure S4). Moreover, the MA isolate showed an occurrence of 1.57 SNPs per 1 Kb found in salt tolerance transcripts, but without any mutation in virulence, temperature, and salinity transcripts (Additional file 1 Figure S1-D). Both NY isolates (a and b) also showed a higher occurrence score of 1.44 and 1.62 SNPs per 1 Kb in salt tolerance transcripts than in virulence transcripts, which was as low as 1.33 and 1.15 SNPs per 1 Kb respectively. It is known that the VA strain and to some extent the NY-a strain, come from an environment that displays low salinity levels [4], while those from Peconic Bay (NY-b) and MA have more stable salinities [5]. Therefore, a higher variants occurrence in environment-related genes could probably be a promoter to enhancing the microbe fitness. In contrast, a low genetic variability in virulence-related genes support that the driver for QPX persistence would be rather the environment, which supports the opportunistic nature of the parasite.

Interestingly the relative abundance of SNPs in virulence-related transcripts was lowest in the VA strains (not considering the MA strain which was used as reference) and highest in the NY-a strain, while diametrically opposing trends were observed in the environment-related transcripts. All of which suggesting a trade-off may exist between local adaptation to environmental conditions and virulence. Nevertheless, ground-truthing the biological significance of the relative distribution of SNPs in virulence and local adaptation genes requires additional targeted experiments contrasting virulence and fitness of various QPX strains under different laboratory conditions.

### Preferential substitution and evolutionary impact

**Fig. 9:**
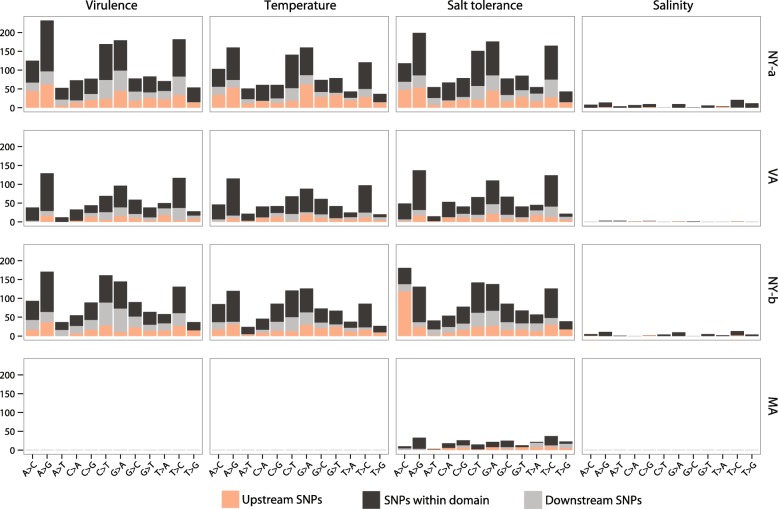
Preferential substitution of SNPs in four QPX strains. Upstream, downstream, and exonic mutations are represented by the number of times they figure in each strain and in each predicted protein

To distinguish which mutations were favored in the four QPX strains, a preferential substitution test was evaluated between strains and domains. On a transcriptome-wide scale, the most represented mutations found were those that changed cytosine nucleobase into thymines (Additional file 1 Figure S5). The second highest were the mutations that changed adenosine nucleobases into guanosines, while the lowest were those that changed thymines into guanosines. The preferential substitution in virulence and local adaptation transcripts was not changed as previously described, however new patterns were slightly more distinguishable (Fig. 9). For example, SNPs were highly concentrated upstream of salt tolerance genes in one of the NY isolate with a preferential substitution of adenosines into cytosines. Lastly, both NY isolates and the VA strain showed preferential substitutions in targeted coding sequences of adenosines into guanosines. With GC content of the QPX parasite being almost 45%, there was a higher chance of forming TT doublets at greater speeds in double stranded DNA [60]. This however did not suggest that recurring thymine dimers in diploid cells were more frequent, usually because of the nature of mixed sequences [61]. In Fig. 5 the MA strain showed a higher homozygosity for both alleles, which presumably was in favor of increasing the chance to form TT doublets. While the biological significance of this pattern is unclear, this information could be relative to the local adaptation of QPX, because cyclobutane pyrimidine dimers (CPD) formed from UV-irradiated cells are prone to DNA lesions and eventually apoptosis [62]. Furthermore, thymine doublets (TT) were previously shown to be involved in a sophisticated mechanism of pushing photoexcited carbon bonds to dimerize and were also responsible in relaxing the ionized state of the DNA and dissipate the radiation as heat, all while protecting the cell [63]. Therefore, the preferential substitutions reported here, especially those found in NY strains could be a leading trend of QPX emergence and its increased fitness level under changing environmental conditions.

## Conclusions

The aim of this study was to examine the evolutionary and functional relatedness of QPX strains originating from several geographical regions of the Northeastern United States. Several QPX strains were selected for genomic screening and the classification of SNPs provided supporting information that a low genotypic diversity in virulence transcripts was a possible complement of a higher adaptation of the parasite in stressful environmental conditions. We propose an overview of the presence of a potential trade-off between virulence and local adaption of the pathogen. Results showed a low mutation rate in QPX virulence genes as compared to environmental-related genes. This low diversity in virulence genes could be an additional indication of the opportunistic nature of the parasite. The analyzed parasitic strains displayed a high diversity in adaptation-related genes such as local temperature and salinity conditions. These results highlighted the adaptation of the parasite to a wide range of environmental conditions and to some extent the spread in North America of some strains under specific environmental conditions, during climate fluctuations, leading to disease outbreaks.

## Additional files

Additional file 1: Supplemental information. A PDF document illustrating additional figures significant for assessing data scalability and pipeline robustness. (PDF 246 kb)

Additional file 2: R implementation. A PDF document with R scripts of descriptive analyses about the data, genomes, sequencing reads, and annotation pipeline. (PDF 5570 kb)

## Abbreviations

QPX: Quahog Parasite Unknown
SNP: Single nucleotide polymorphism
NY-a: New York QPX strain a
NY-b: New York QPX strain b
MA: Massachusetts
VA: Virginia
GATK: Genomic Analysis Toolkit
Kb: Kilo (thousand) base pair
Mbp: Mega (million) base pair
TT: Thymine doublets
