# Supplementary material for: SNP hot-spots in the clam parasite QPX
Source: BMC Genomics. 2018 Jun 20;19:486. doi: 10.1186/s12864-018-4866-8 (PMC6011583; doi:10.1186/s12864-018-4866-8)
Supplement: Supplementary file 1 — Supplemental information. A PDF document illustrating additional figures significant for assessing data scalability and pipeline robustness. (PDF 246 kb) [file 12864_2018_4866_MOESM1_ESM.pdf]

Supplemental information

SNP hot-spots in the clam parasite QPX

The data we have include 4 QPX transcriptomes generated from four strains of the parasite. Our aim is to comprehensively examine their evolutionary and functional relatedness. We also acquired a published draft of the QPX genome.

- There are multiple levels of host resistance and pathogen virulence.
- Hypothesis 1: QPX is genetically diverse allowing the parasite to flourish in various environmental conditions.
- Hypothesis 2: Trade-off exists between virulence and local adaption of the pathogen.
- Compare four QPX strains (transcriptomes) that derive from different geographical locations
- First goal is to study the relatedness between strains (or closeness using SNP data)
- Hierarchical clustering was thus used for strain relatedness.
- Preliminary results were significant.
- Additional analyses were done on variant calling.
- Differential distribution of SNPs and indels was observed between strains.
- Preferential substitution of SNPs was observed between strains.
- Contigs were annotated with Pfam domains.
- Differential distribution of SNPs inside and outside domains were investigated.
- Higher number of variants were found outside of virulence protein domains.
- Higher number of variants were found inside salinity protein domains.

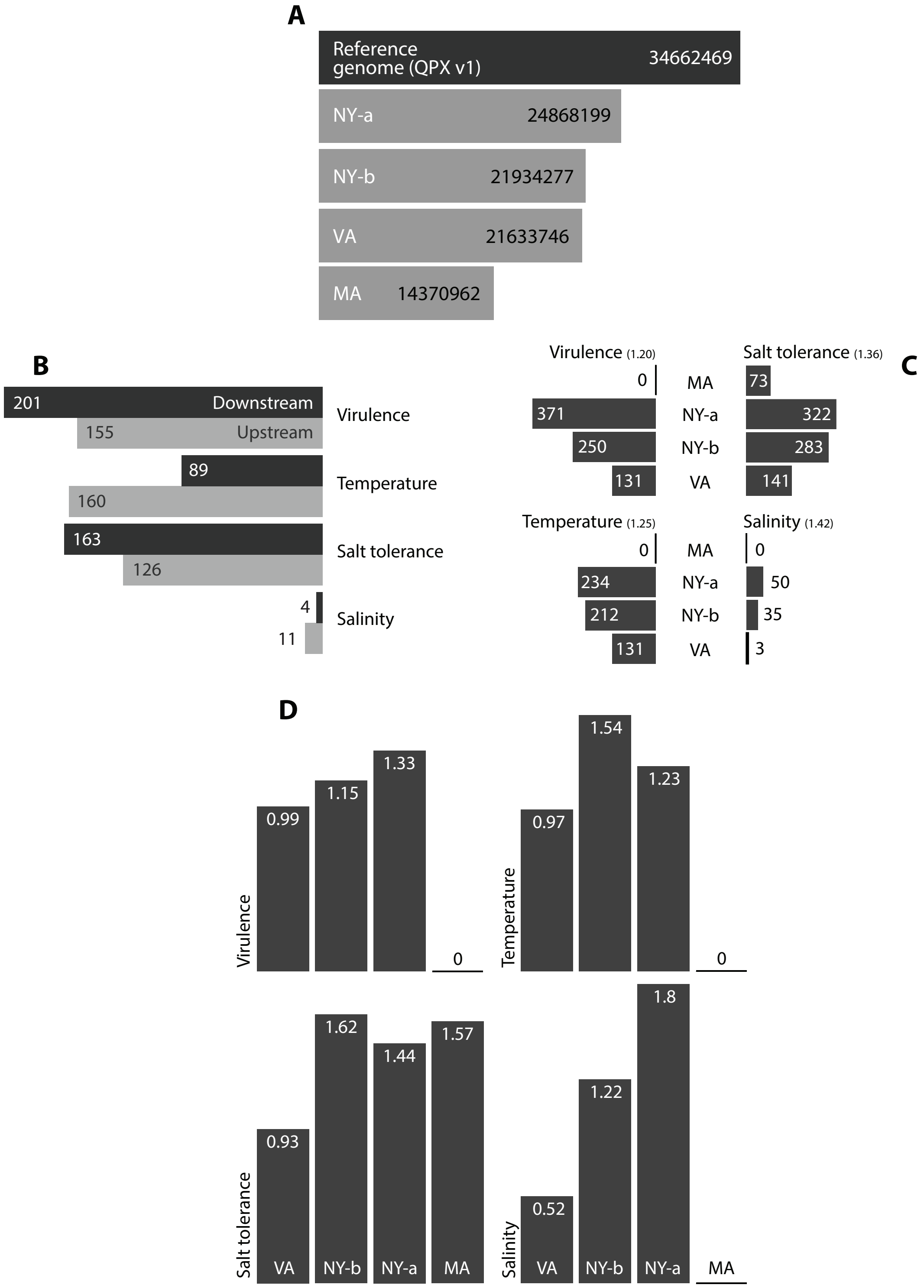

Figure S1: Summary of contig counts, Pfam domains, and SNP called in four QPX strains QPX libraries used are those of New York (NY), Massachusetts (MA), and Virginia (VA). Contig annotation at e-value $\leq 10^{-10}$ and SNP calling after two recalibrations. A- Number of sequenced bases for each QPX library. B- Standardized number of SNPs outside protein domains per unique Pfam accession number. C- Number of SNPs within domains per QPX strain and per virulence or adaptation genes. D- Frequency of SNPs within protein domains in four QPX strains. Each frequency is the SNP count per strain per Pfam category normalized by the total sum of the length of QPX contigs for each strain.

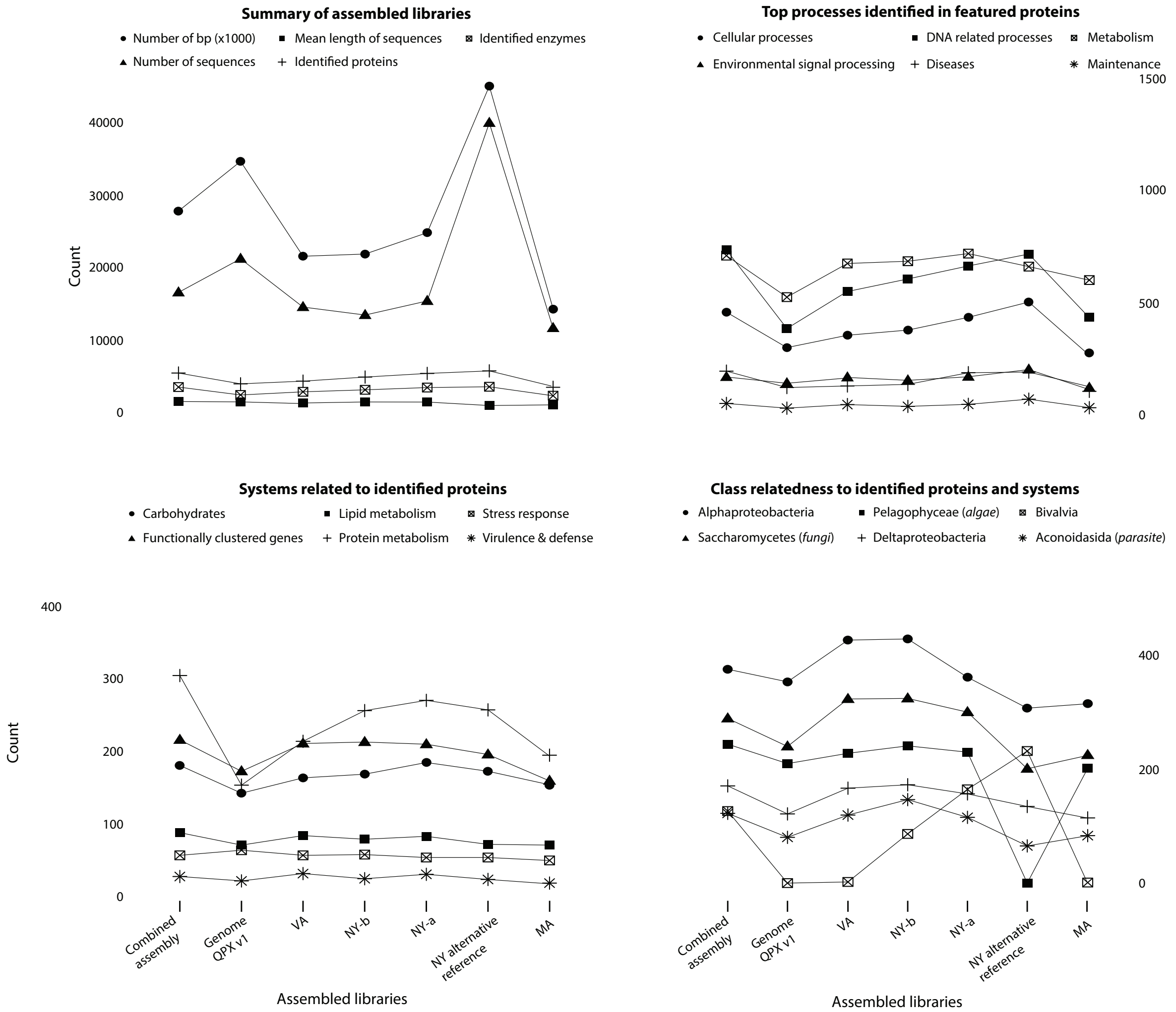

Figure S2: Descriptive analysis of all QPX isolates by fast annotation of contigs All four QPX libraries are assembled into contigs and fast annotated for a functional analysis. QPX libraries used are those of New York (NY), Massachusetts (MA), and Virginia (VA). Scores from predicted protein features and phylogenetic classification of contigs are used for a comprehensive comparison between samples. We also included contigs relative to three references, i- Combined reference: includes an assembly of all QPX libraries merged together, ii- genome reference of QPX v1 (Figure 2), and iii- custom assembly of NY library as a reference transcriptome (NY alternative reference).

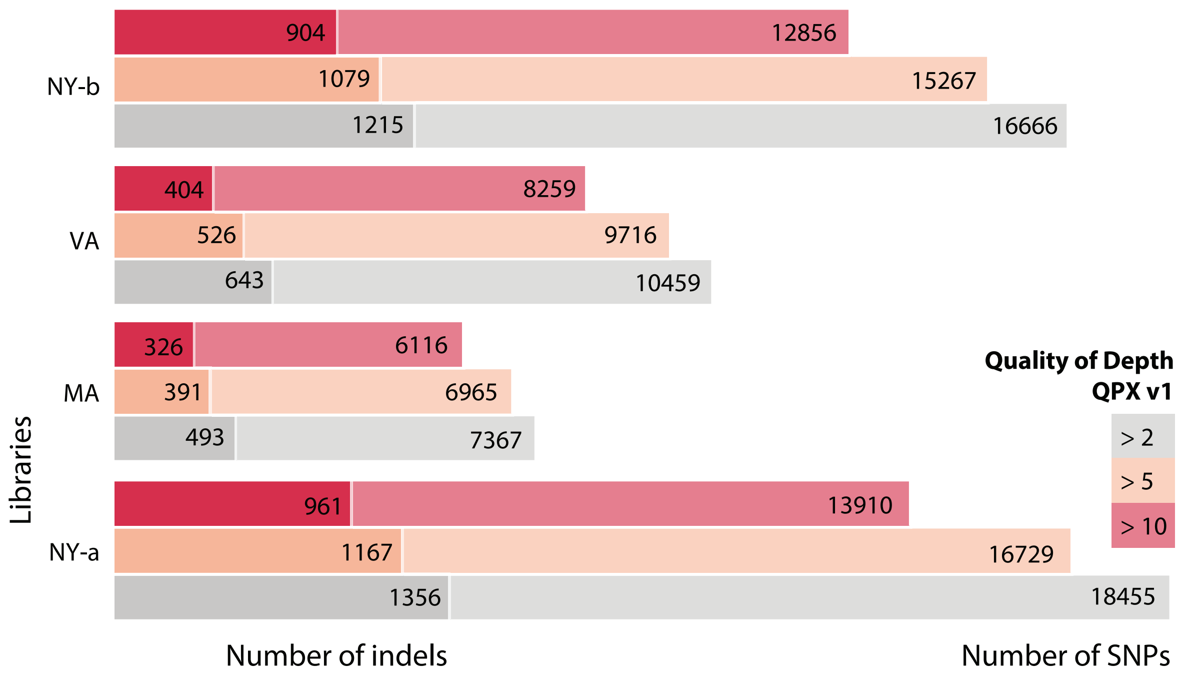

Figure S3: Difference in indel and SNP counts for all QPX isolates with reference genome QPX v1 Base-calling is done with the reference genome QPX v1. QPX libraries used are those of New York (NY), Massachusetts (MA), and Virginia (VA).

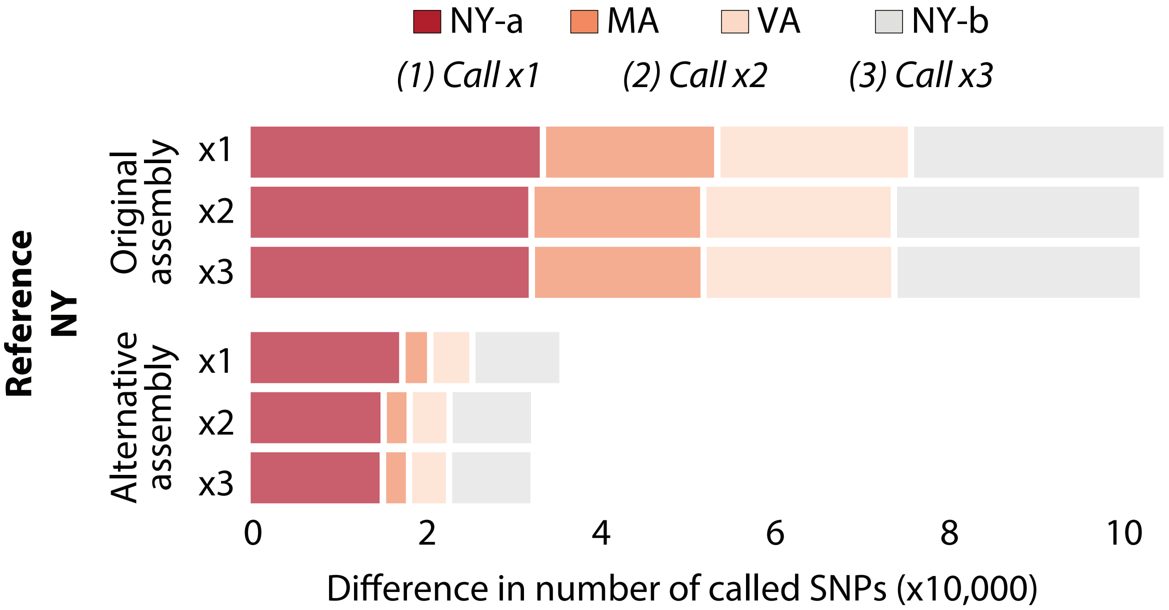

Figure S4: Difference in SNP count from GATK recalibration-filtering protocol GATK is used for base-calling. Here we show the use of GATK on two reference transcriptomes, both based on the NY-a strain. References are assembled here with different options, relaxed thresholds (original) and stringent thresholds (alternative). The parameters used here are also used for base-calling with the QPX v1 reference (Figure 2) in the final SNP analyzes. QPX libraries used are those of New York (NY), Massachusetts (MA), and Virginia (VA). Calls (x1, x2, x3) represent the number of recalibration-filtering runs in GATK.

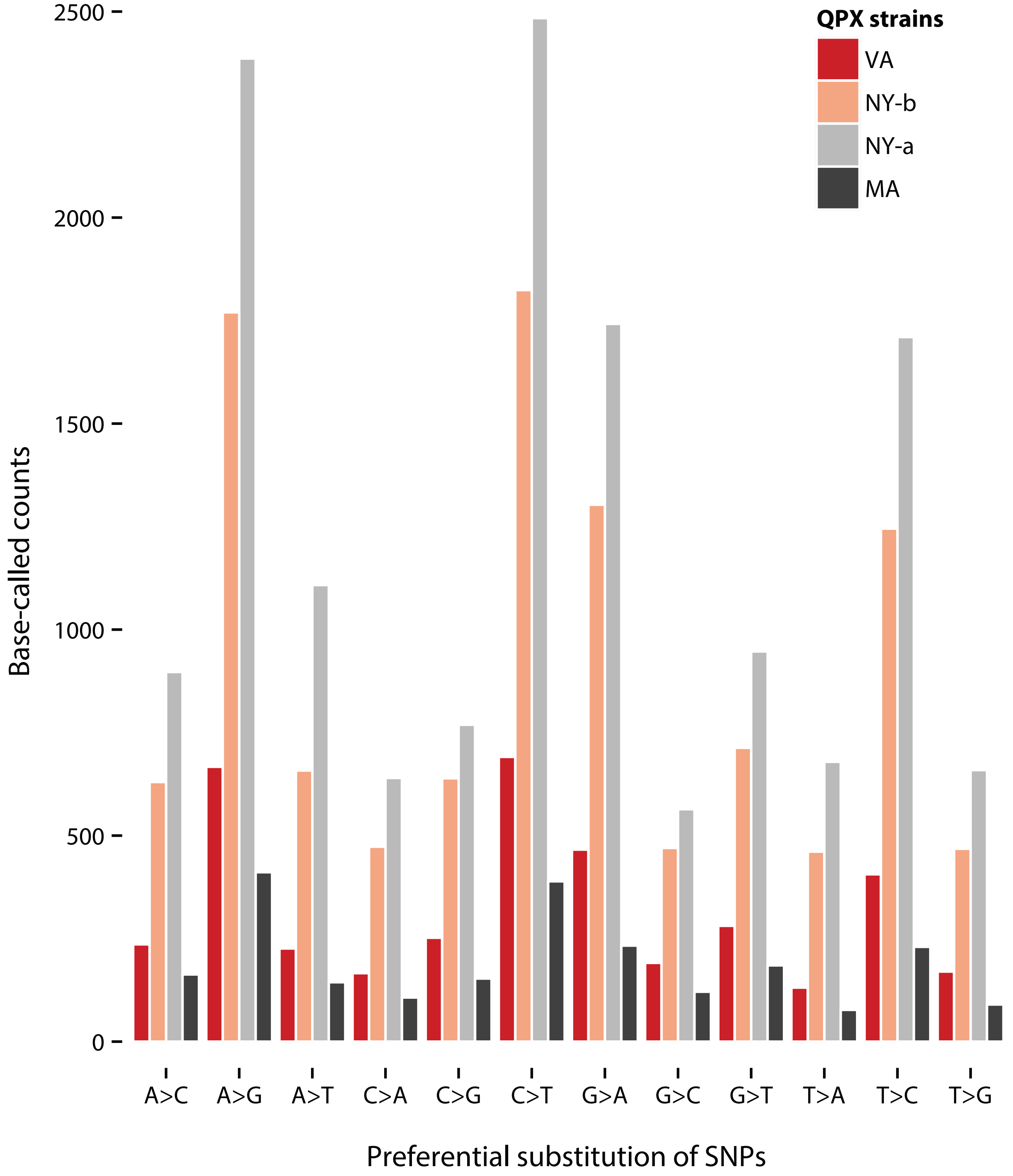

Figure S5: Transcriptome-wide preferential substitution of SNPs in four QPX strains QPX libraries used are those of New York (NY), Massachusetts (MA), and Virginia (VA). GATK is used for base-calling.
