# Supplementary material for: SNP hot-spots in the clam parasite QPX
Source: BMC Genomics. 2018 Jun 20;19:486. doi: 10.1186/s12864-018-4866-8 (PMC6011583; doi:10.1186/s12864-018-4866-8)
Supplement: Supplementary file 2 — R implementation. A PDF document with R scripts of descriptive analyses about the data, genomes, sequencing reads, and annotation pipeline. (PDF 5570 kb) [file 12864_2018_4866_MOESM2_ESM.pdf]

R implementation

Sleiman Bassim

September 11, 2017

Loaded functions:

```
#source("/media/Data/Dropbox/humanR/01funcs.R")
rm(list=ls())
```

Load packages.

```
pkgs <- c('gdata','lattice','latticeExtra',
          'ggplot2', 'dplyr', 'vegan', 'tidyr',
          'ggbiplot')
lapply(pkgs, require, character.only = TRUE)
```

1 Show the length distribution of reads

Load *gff3* sequence length data for mapped QPX libraries and references. GFF3 files contain the sequence length of each contig. These contigs belong to Steve Roberts genome v015 and 017 and transcriptome v21 of QPX.

↰ Refer to github front page of the rnaseQPX project

```
genome <- read.table("./data/QPX_Genome_v017.gff3")
head(genome)

                     V1 V2  V3 V4    V5 V6 V7 V8
1 QPX_v017_contig_1007  . CDS  1 15433  .  .  .
2 QPX_v017_contig_1043  . CDS  1 11565  .  .  .
3 QPX_v017_contig_1050  . CDS  1 12908  .  .  .
4 QPX_v017_contig_1087  . CDS  1 12852  .  .  .
5 QPX_v017_contig_1094  . CDS  1 10365  .  .  .
6 QPX_v017_contig_1128  . CDS  1 10580  .  .  .
                                         V9
1 ID=QPX_v017_contig_1007;Name=QPX_v017_contig_1007
2 ID=QPX_v017_contig_1043;Name=QPX_v017_contig_1043
3 ID=QPX_v017_contig_1050;Name=QPX_v017_contig_1050
4 ID=QPX_v017_contig_1087;Name=QPX_v017_contig_1087
5 ID=QPX_v017_contig_1094;Name=QPX_v017_contig_1094
6 ID=QPX_v017_contig_1128;Name=QPX_v017_contig_1128

transcriptome <- read.table("./data/QPX_transcriptome_v2orf.gff3")
```

GFF3 counts of MME transcriptomes MMETSP0098 and MMETSP00992, and the custom assembly with MMETSP0098.

```
mme98 <- read.table("./data/MMETSP0098.gff3")
mme99 <- read.table("./data/MMETSP0099_2.gff3")
mme98c <- read.table("./data/mme98cust.gff3")
genomv015 <- read.table("./data/QPX_v015.gff3")
```

The number of bases has been counted and published elsewhere by the authors who assembled the references and sequenced the QPX libraries. Working through their data, we provide a distribution of contig length for genome of Steve's QPX. The purpose of this analysis is to identify 2 things:

- Biases in contig length
- Comparison of parameters used for assembling the references

```r
histogram(~ (genome$V5),
          type= 'count',
          nint = 75,
          data = genome,
          xlab = 'Sequence length (bp)',
          ylab = 'Nb of contigs (555 total)',
          col = 'red')
```

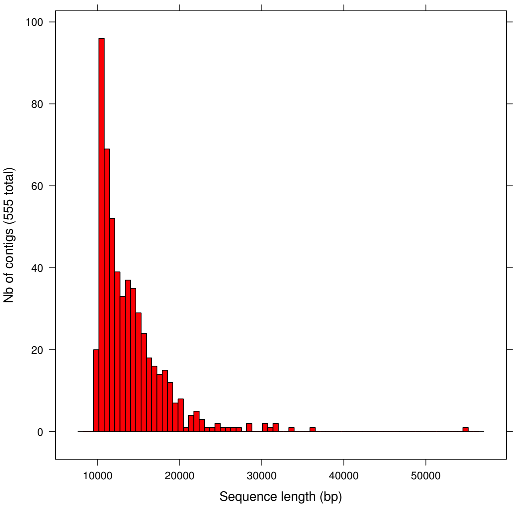

Distribution of Steve's QPX transcriptome.

```r
histogram(~ transcriptome$V5,
          type = 'count',
          col = 'red',
          data = transcriptome,
          nint = 75,
          xlab = 'Sequence length (bp)',
          ylab = 'Nb of contigs (11774 total)')
```

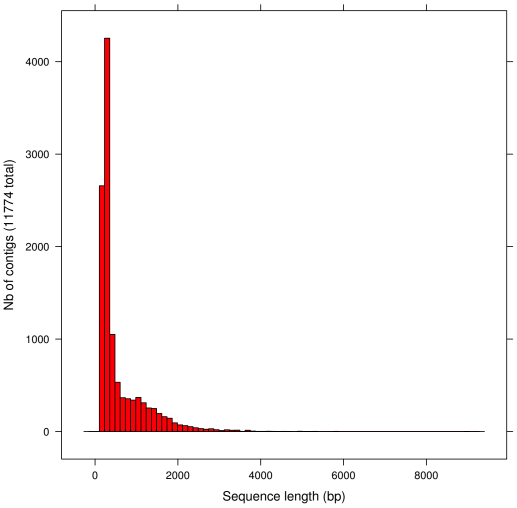

Distribution of length of MMETSP0098.

```r
histogram(~ mme98$V5,
          type = 'count',
          nint = 75,
          data = mme98,
          xlab = 'Sequence length (bp)',
          ylab = 'Nb of contigs (11774 total)')
```

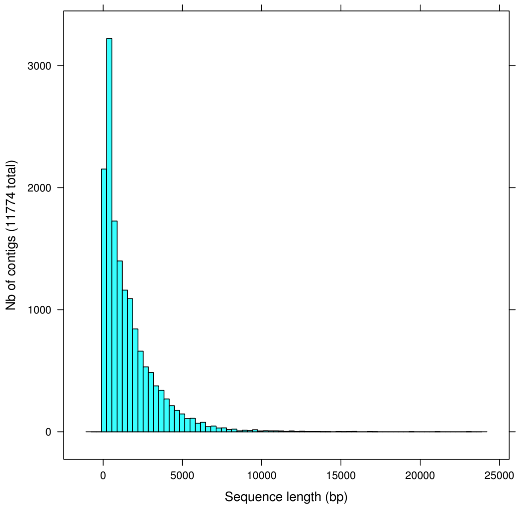

Superpose the length of contigs in:

- Steve's genome v017 (555 contigs)
- Steve's transcriptome
- MMEtsp0098 transcriptome
- MMEtsp00992 transcriptome
- MMEtsp0098 custom transcriptome
- Steve's Genome v015 (approx 22,000 contigs)

Merge datasets.

↰ Assign a new column to identify contigs.

```
grouping <- rbind(genome[, c(1,5)],
                  transcriptome[, c(1, 5)],
                  mme98[, c(1,5)],
                  mme99[, c(1,5)],
                  mme98c[, c(1,5)],
                  genomv015[, c(1,5)])
grouping <- data.frame(grouping,
                       y = c(rep("GenomeV17", nrow(genome)),
                           rep("TrxV22", nrow(transcriptome)),
                           rep("MME98", nrow(mme98)),
                           rep("MME99", nrow(mme99)),
                           rep("MME98custom", nrow(mme98c)),
                             rep("(GenomeV15)", nrow(genomv015))))

dim(grouping)

[1] 100811      3
```

Plot reads length of the 6 assemblies including 2 QPX genomes.

↰ A higher resolution of this chart can be found in the Supplemental Information

```
custom.colors <- c(col1 = "#762a83",
```

```
                    col2 = "#1b7837",
                    col3 = "#ef8a62",
                    col4 = "#2166ac",
                    col5 = "#8c510a",
                    col6 = "#e6ab02")

histogram( V1 ~ V5,
     data = grouping,
     nint = 55,
     scales = list(log = 10),
     type = "p",
     #breaks = seq(4,8,by=0.2),
     ylim = c(0,28),
     groups = grouping$y,
     panel = function(...) panel.superpose(...,
         panel.groups = panel.histogram,
         col = custom.colors,
         alpha = 1),
     auto.key=list(columns=3,
         rectangles = FALSE,
         col = custom.colors),
     main = 'Different QPX assemblies and the length of their contigs',
     xlab = 'Length of all contigs',
     ylab = 'Percentage of the total assembly (count)'
     )

Warning in histogram.formula(V1 ~ V5, data = grouping, nint = 55, scales = list(log = 10),
:  Can't have log Y-scale
```

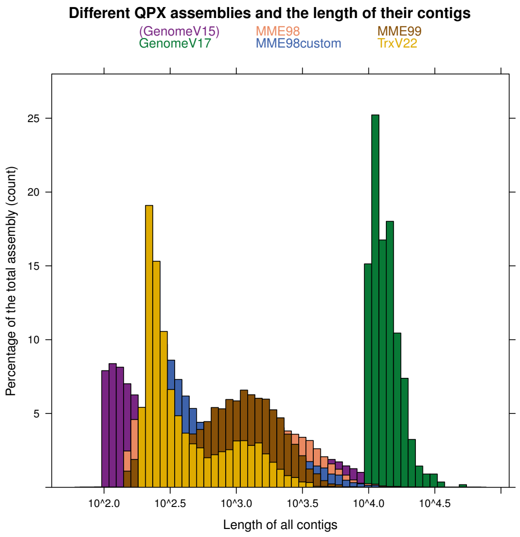

2 Calling SNPs: Testing tools, parameters, and filters

SNPs were called either with samtools *mpileup* function and the highest significant were selected with bcftools or they have been called with GATK. Either way SNP calling was done on each QPX library separately. QPX libraries were:

- mmetsp0098 from New York
- mmetsp001433 from New York
- mmetsp00992 from Massachusetts
- mmetsp001002 from Virginia
- mmetsp0099 from Massachusetts
- mmetsp00100 from Virginia

2.1 Load data

Number of SNPs called with either packages were counted. Calls were done after read duplicates were removed with Picard.

```
counts.SNP <- read.xls("./data/snp.counts.xlsx", sheet = 1)
glimpse(counts.SNP)

Observations: 114
Variables: 3
$ sample    (int) 98, 992, 1002, 1433, 99, 100, 98, 992, 1002, 14...
$ counts    (int) 351790, 395060, 427790, 389188, 309813, 425947,...
$ reference (fctr) trxSRv21, trxSRv21, trxSRv21, trxSRv21, trxSRv...
```

Histogram grouped by QPX library showing difference in SNPs counts relative to the reference used for mapping and the number of times GATK has been used to recalibrate calls. GATK (x1, x2, x3) represent one, two or three rounds of recalibration. The recalibration is done over a list of variants called under stringent parameters. SR: Steve Roberts genomes. Cust: custom assembly of mmetsp0098.

↰ Recalibration is done with GATK. The strategy is described in the pipeline on github here.

```
xyplot( factor(reference) ~ as.matrix(counts) | factor(sample),
       data = counts.SNP[-c(1:24), ],
       groups = counts.SNP$reference,
       pch = 21,
       cex = 1,
       type = c("p"),
       xlab = 'Number of SNPs called',
       ylab = 'References & GATK filters')
```

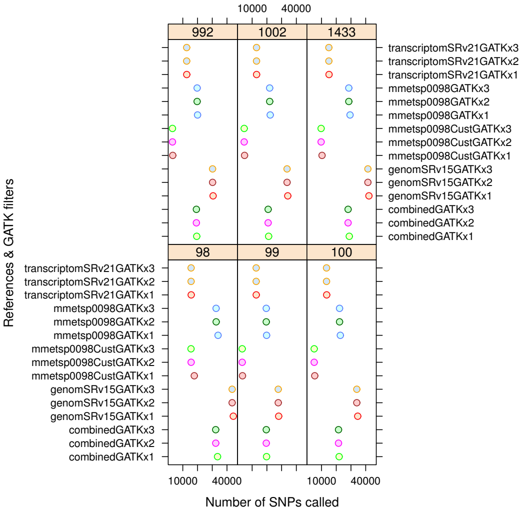

Plot the difference between QPX libraries and variant calling packages for the number of called SNPs. Combined: an assembly made of the combination of mmetsp0098, 992, 1002, 1433. SR: Steve Roberts genomes and transcriptomes (15 and 21 respectively). Samtools was done on trxSRv21rmdup.

```
ggplot(counts.SNP,
       aes(x = factor(sample),
           y = counts,
           fill = factor(reference))) +
    geom_bar(stat = "identity",
             position = "dodge") +
        theme_bw() +
    labs(title = "SNP counts between GATK and samtools with 6 references",
         x = "QPX Libraries",
         y = "SNP count")
```

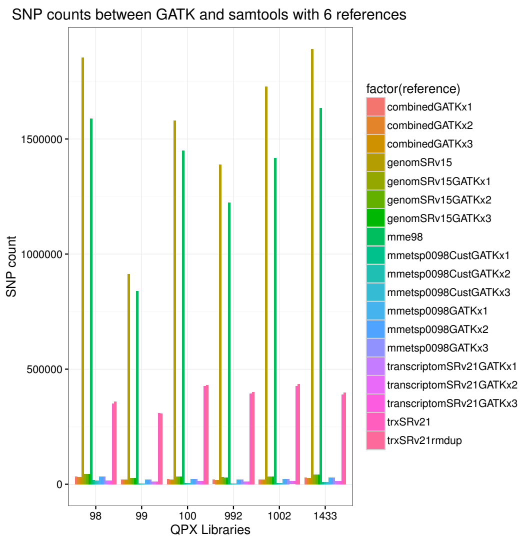

Another plot for cluster analysis between references and SNPs called. I find this useful for a fast check of outliers and errors in importing data. In *comG3* com=combined assembly, G=genome reference, 3= x3 recalibration rounds.

```
dat <- read.xls("./data/snp.counts.xlsx", sheet = 4)
custom.colors <- c(col1 = "#b2182b",
                   col2 = "#ef8a62",
                   col3 = "#fddbc7",
                   col4 = "#e0e0e0",
                   col5 = "#999999",
                   col6 = "#4d4d4d")
barplot((as.matrix(dat[, -c(1:5)])),
        col = custom.colors,
        horiz = TRUE,
        las = 2,
        beside = T,
        legend.text = factor(dat[, 1]),
        cex.names = .7,
        xlab = '(log10) Number of SNPs called',
        ylab = 'References')
```

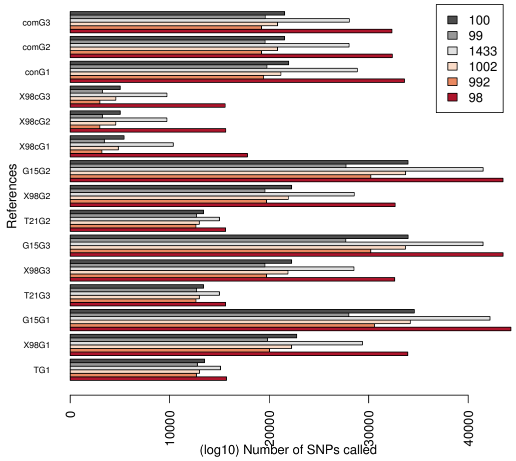

Plotting only the GATK called SNPs.

```
counts.SNP <- counts.SNP[-c(1:24), ]
```

```r
ggplot(counts.SNP,
       aes(x = factor(sample),
           y = counts,
           fill = factor(reference))) +
    geom_bar(stat = "identity",
             position = "dodge") +
        theme_bw() +
    labs(x = "QPX Libraries",
         y = "SNP counts")
```

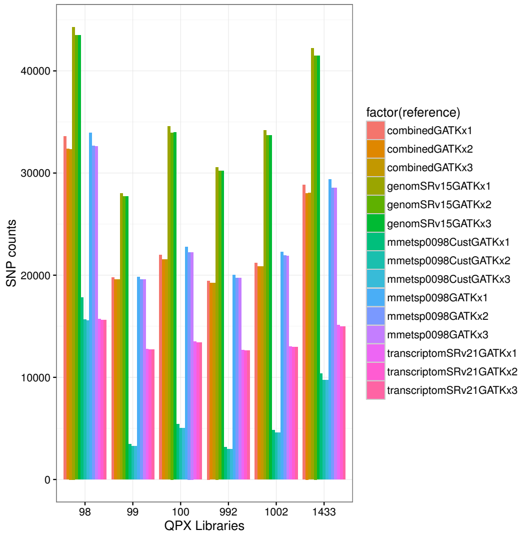

Plot the difference between the number of SNPs called on the 6 QPX libraries using either the assembled or custom assembled mmetsp0098 reference. Also show the variation pattern with the number of reads used for calling SNPs. First, prepare SNP data.

```r
x1 <- counts.SNP[counts.SNP$reference %in% "mmetsp0098GATKx1", ]
x2 <- counts.SNP[counts.SNP$reference %in% "mmetsp0098CustGATKx1", ]
```

Next, add the number of reads per QPX library. This is the count of non duplicate reads that mapped to each of all the references used.

```r
ref.reads <- read.xls("./data/refreads.xlsx", sheet = 1)
head(ref.reads)

  sample  counts        reference
1     98 8591456 mmetsp0098GATKx1
2    992 5875110 mmetsp0098GATKx1
3   1002 7780584 mmetsp0098GATKx1
4   1433 7001081 mmetsp0098GATKx1
5     99 4835298 mmetsp0098GATKx1
6    100 4193326 mmetsp0098GATKx1

y <- ref.reads[1:12, ]
```

Plot difference.

```r
dat <- data.frame(rbind(x1, x2), reads = y$counts)
```

```
ggplot(dat,
       aes(x = factor(sample),
           y = counts,
           fill = factor(reference))) +
    geom_bar(stat = "identity",
             position = "dodge") +
        theme_bw() +
    labs(x = "QPX Libraries",
         y = "SNP counts")

ggplot(dat,
       aes(x = factor(sample),
           y = reads,
           group = factor(reference))) +
    geom_line(size = .2)+
        geom_point(data = dat,
                   aes(x = factor(sample),
                       y = reads,
                       colour = factor(reference),
                       size = counts)) +
        theme_bw() +
        labs(x = "QPX Libraries",
             y = "Mapped reads counts")
```

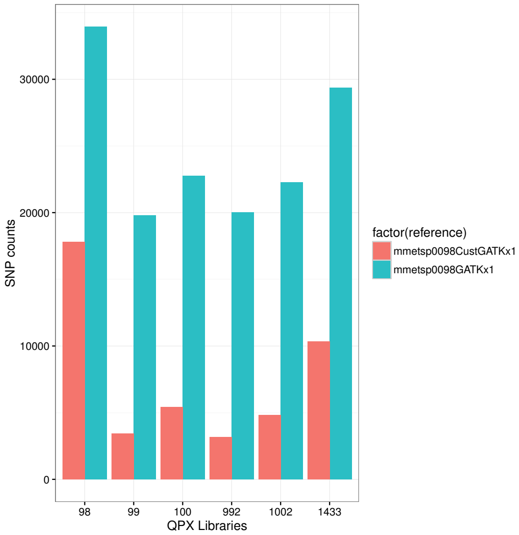

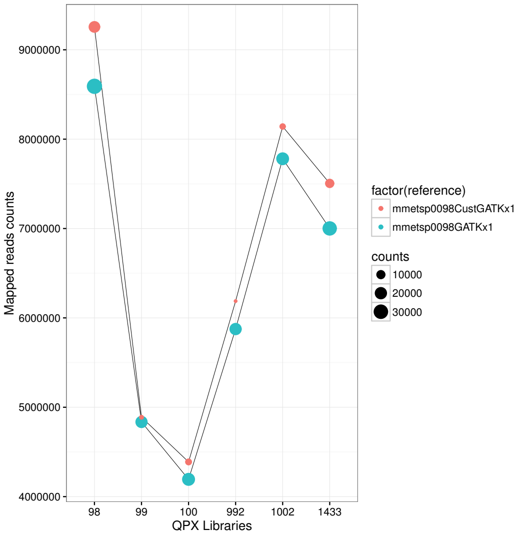

Plot number of all mapped reads for each QPX library and for all 4 references.

```
ggplot(ref.reads,
       aes(x = factor(sample),
           y = counts,
           group = factor(reference))) +
    geom_line(size = .2) +
        geom_point(aes(shape = factor(reference))) +
            theme_bw() +
        labs(x = "QPX libraries",
             y = "Read counts")
```

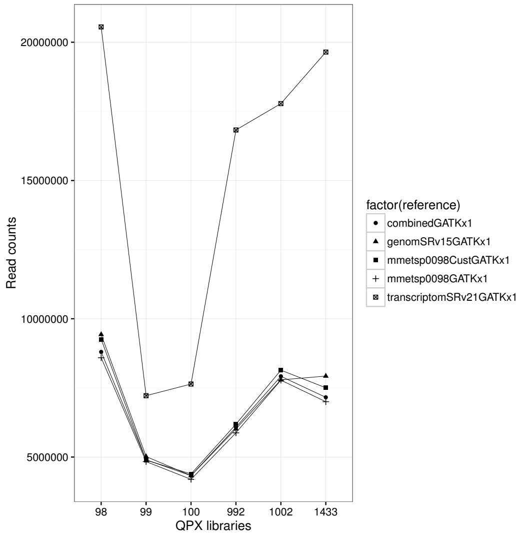

Difference in SNPs called between the already assembled and the custom assembled *mmetsp0098* reference.

↰ The custom assembled mmetsp0098 was done with trinity

```
dat <- read.xls("./data/snp.counts.xlsx", sheet = 2)
ggplot(dat,
       aes(x = factor(sample),
           y = counts,
           fill = factor(reference))) +
    geom_bar(stat = "identity",
             position = "dodge") +
        theme_bw() +
    labs(x = "QPX libraries",
         y = "SNP counts")
```

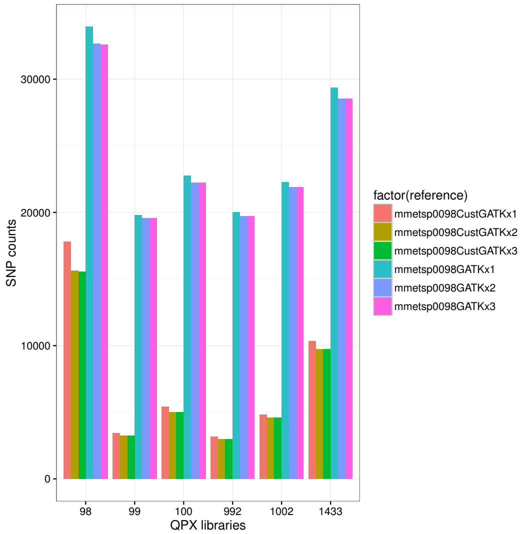

Another way to show difference between GATK recalibration protocols and the way this strategy decreases the number of SNPs called by readjusting of nucleotide probabilities for each read. The plot shows also the difference in SNP called between mmetsp0098 custom (the first 3 bars) and original (the last 3 bars) assemblies.

```
dat <- read.xls("./data/snp.counts.xlsx", sheet = 3)
```

```r
custom.colors <- c(col1 = "#b2182b",
                   col2 = "#ef8a62",
                   col3 = "#fddbc7",
                   col4 = "#e0e0e0",
                   col5 = "#999999",
                   col6 = "#4d4d4d")

barplot(as.matrix(dat[, -1]),
        horiz = TRUE,
        col = custom.colors,
        xlab = "Difference in number of called SNPs between references",
        ylab = 'Difference in GATK filtering protocols',
        las = 2,
        legend = dat$sample)
```

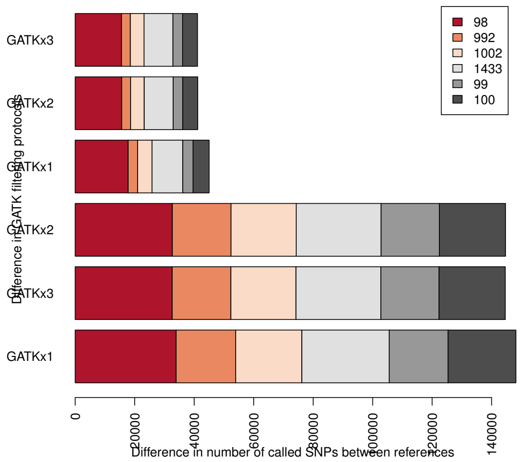

3 Final filtering

GATK hard filtering removes SNPs with low quality or confidence. This is calculated relatively to the depth of coverage. Using 3 three different thresholds for *QD* (qualtiy of depth) we get the number of SNPs that pass the filters.

$$QD = \frac{Confidence}{DepthCoverage} \tag{1}$$

$$DepthOfCoverage = \frac{NbOfReads \times ReadLength}{AssemblySize} \tag{2}$$

3.1 Working with the combined assembly and genome v15

Difference in called SNPs between QPX libraries mapped to 2 different references, the combined assembly (represented by lower bar labels) and the genome v15 of S. Roberts (represented by higher bar labels). *A higher resolution bar can be found in Supplemental Information.*

↰ Genome v15 S. Roberts is used in the remaining tests

```r
dat <- read.xls("./data/hard.snps.xlsx", sheet = 1)
```

```
ggplot(dat,
       aes(x = factor(sample),
           y = snps,
           fill = factor(qd)
#            group = factor(reference)
           )) +
    geom_bar(stat = "identity",
             position = "dodge") +
        theme_bw() +
            geom_text(aes(x = factor(sample),
                          y = snps,
                          ymax = snps,
                          label = snps,
                          size = 2,
                          hjust = 1),
                      position = position_dodge(width=1)) +
                coord_flip() +
                    scale_fill_brewer() +
                labs(x = "Count of SNPs ref:combined (lower) and ref:genome15 (higher)",
                     y = "QPX libraries")
```

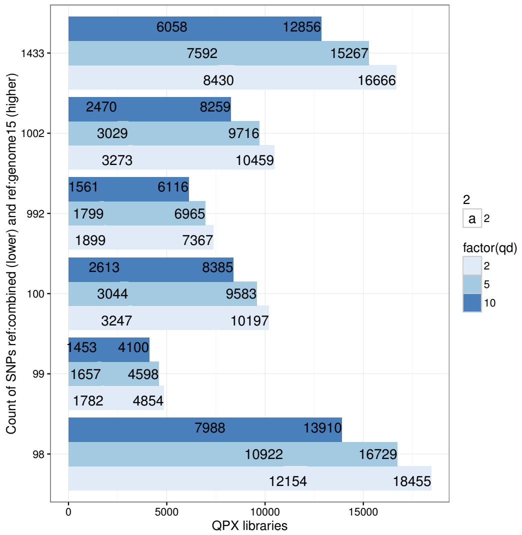

Number of SNPs per strain at *QD = 5*. SNPs called against *SR genome v15*. The number of reads (approx 100 nt in size) per library has been counted and plotted previously, the data is in *refreads.xlsx*.

```
dat <- read.xls("./data/hard.snps.xlsx", sheet = 1)
dat <- dat[7:10, 1:2]
dat$Treads <- ref.reads[c(19, 22, 20, 21), 2]
dat$norm <- with(dat, (snps/Treads)*1000)

ggplot(dat,
       aes(x = as.factor(sample),
           y = norm)) +
    geom_bar(stat = "identity") +
        geom_text(aes(x = as.factor(sample),
                      y = norm,
                      ymax = norm,
                      label = round(norm, digits = 2),
                      color = "white",
                      vjust = 2,
                      size = 3)) +
        labs(x = "QPX libraries",
             y = "Normalized count of nb of SNPs/nb of reads")
```

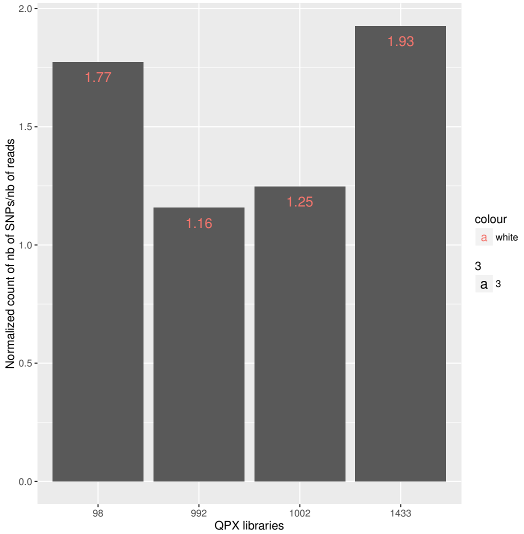

We can also do the same thing with indels.

```
dat <- read.xls("./data/hard.snps.xlsx", sheet = 1)
ggplot(dat,
       aes(x = factor(sample),
           y = indels,
           fill = factor(qd))) +
    geom_bar(stat = "identity",
             position = "dodge") +
        theme_bw() +
            geom_text(aes(x = factor(sample),
                          y = indels,
                          ymax = indels,
                          label = indels,
                          size = 2,
                          hjust = 1),
                     position = position_dodge(width=1)) +
                   coord_flip() +
                       scale_fill_hue(c = 40, l = 60) +
                   labs(x = "Count of indels ref:combined (lower), ref:genome15 (higher)",
                        y = "QPX libraries")
```

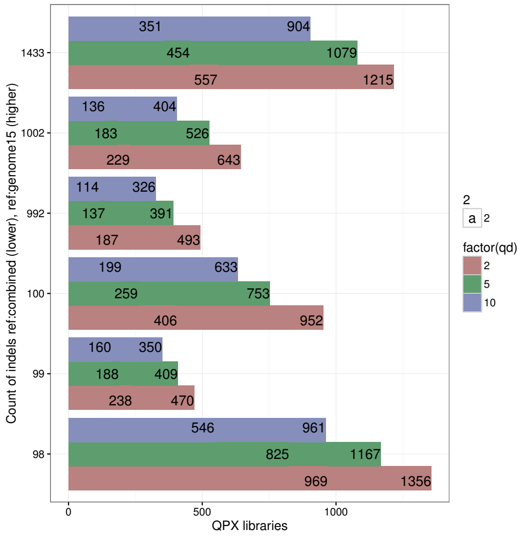

The combined assembly is already published. It is added here with the other references because it is heavily annotated and their contigs are extensively mapped. Load in new mapped data to the combined reference:

```
combined <- read.xls("./data/snp.counts.xlsx", sheet = 1)
glimpse(combined)

Observations: 114
Variables: 3
$ sample    (int) 98, 992, 1002, 1433, 99, 100, 98, 992, 1002, 14...
$ counts    (int) 351790, 395060, 427790, 389188, 309813, 425947,...
$ reference (fctr) trxSRv21, trxSRv21, trxSRv21, trxSRv21, trxSRv...
```

Difference in SNPs called between the genome v15 of S. Roberts and the official combined assembly. First extract relative rows.

```
dev <- paste("genomSRv15GATKx", seq(1,3,1), sep = "")
ser <- paste("combinedGATKx", seq(1,3,1), sep = "")
difference <- rbind(combined[combined$reference %in% dev, ],
               combined[combined$reference %in% ser, ])

d.ref <- ref.reads[c(19:30), ]
```

Plot difference.

↰ A higher resolution of this plot can be found in supplemental Information

```
ggplot(difference,
       aes(x = factor(sample),
           y = counts,
           fill = factor(reference))) +
    geom_bar(stat = "identity",
             position = "dodge") +
        theme_bw()

ggplot(d.ref,
       aes(x = factor(sample),
           y = counts,
           group = factor(reference))) +
    geom_line(size = .2)+
        geom_point(data = d.ref,
                   aes(x = factor(sample),
                       y = counts,
                       colour = factor(reference),
                       size = counts)) +
        theme_bw()
```

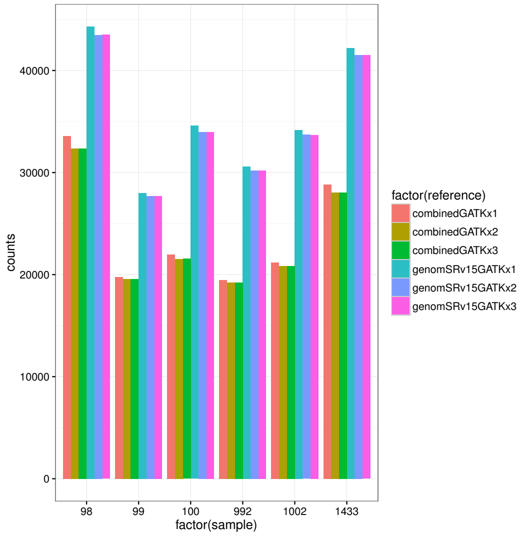

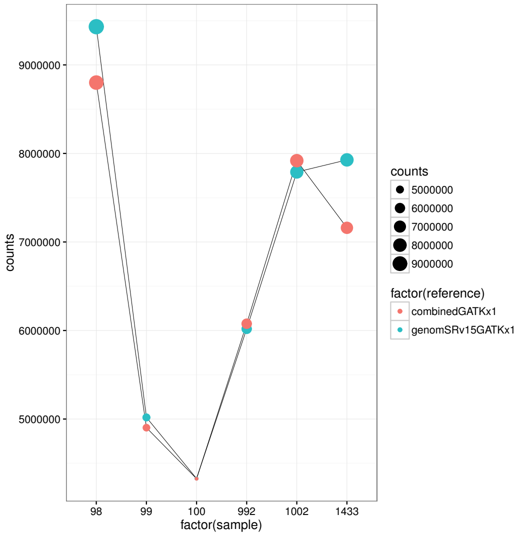

4 Descriptive stats of all processed libraries

This following section shows the mean length of all sequences assembled from each library, the number of base pairs per library, the identified protein features from these sequences, and the number of functional enzymes identified by mapping to public libraries. It is to note the number of predicted and identified rRNA features in each of these libraries is significantly low. Regress different variables on each others for visualization purposes.

```
stats <- read.xls("./data/libraries.xlsx", sheet = 1)
rstats <- stats[complete.cases(stats), ]
#rownames(rstats) <-  stats[, 1]

# The whole new magical script
# job: order columns
# dependecies: dplyr
rstats <- within(rstats,
                 libraries <- factor(libraries,
                                     levels = arrange(rstats,
                                         bp)$libraries))
```

```
Warning:  failed to assign NativeSymbolInfo for env since env is already defined in the 'lazyeval'
namespace
```

```
ggplot(rstats,
       aes(x = libraries,
           y = bp)) +
    geom_bar(stat = "identity") +
        theme_bw() +
            coord_flip() +
                geom_text(aes(x = libraries,
                              y = bp,
                              ymax = bp,
                              label = bp,
                              size = 5,
                              color = "white",
                              hjust = 1.2)) +
                labs(x = "Number of base pair (bp) in the sequenced QPX libraries",
                     y = "QPX libraries")
```

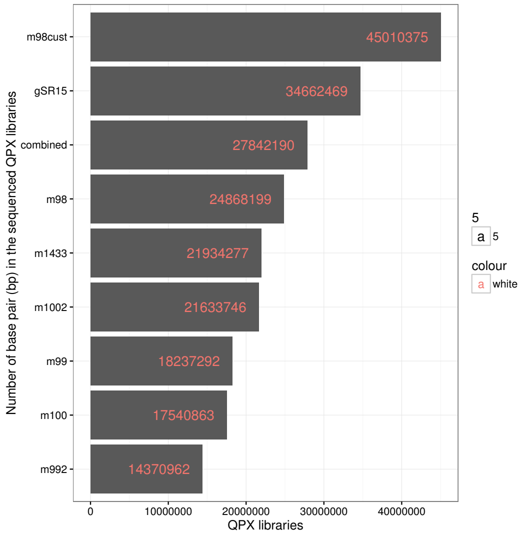

The plot shows the difference between QPX libraries according to the number of base pair (bp x 1000, the identified protein features estimated from assembled sequences (feature), the functional sequences estimated from the contigs (function), the mean length of contigs in each library, and the number of contigs (sequence) assembled from raw reads after trimming and duplicate removal (all basic quality controls).

```
stats <- read.xls("./data/libraries.xlsx", sheet = 1)
```

↰ The number of base pair must be multiplied by 1000 in this chart

```r
rstats <- stats[complete.cases(stats), ]
rstats$bp <- rstats$bp/1000
#rstats <- rename(rstats, bpx1000 = bp)
rstats <- gather(rstats, "feature", "count", c(2:4, 7:8))
ggplot(rstats,
       aes(x = libraries,
           y = count,
           group = factor(feature))) +
    geom_line(size = .2) +
        geom_point(aes(shape = factor(feature),
                           size = 1.5)) +
                           theme_bw() +
                               geom_text(aes(x = libraries,
                                             y = count,
                                             ymax = count,
                                             label = count,
                                             size = 1.5,
                                             hjust = ifelse(sign(count)>1, .5, 0)),
                                         position = position_dodge(width = 1)) +
                               labs(x = "QPX libraries",
                                    y = "Counts")
```

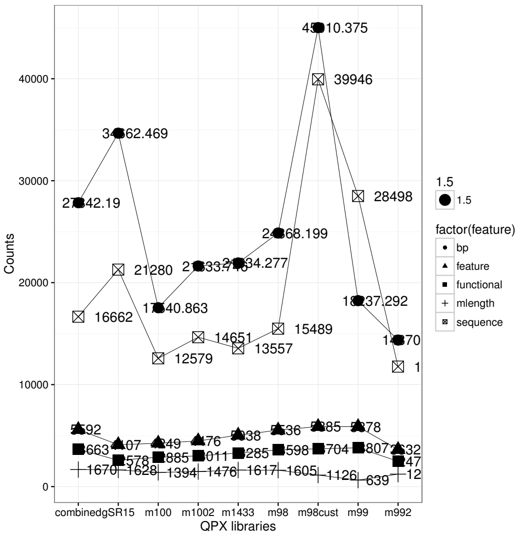

Principal component analysis and diagnostics.

```r
stats <- read.xls("./data/libraries.xlsx", sheet = 1)
rownames(stats) <- stats$libraries
rstats <- stats[complete.cases(stats), -1]
rstats <- decostand(rstats, method = "range")
p = princomp(~bp + mlength + sequence + rna
   , data= rstats)
summary(p)

Importance of components:
                       Comp.1 Comp.2 Comp.3   Comp.4
Standard deviation      0.459  0.382 0.1401 0.017928
Proportion of Variance  0.560  0.387 0.0522 0.000855
Cumulative Proportion   0.560  0.947 0.9991 1.000000

biplot(p)
```

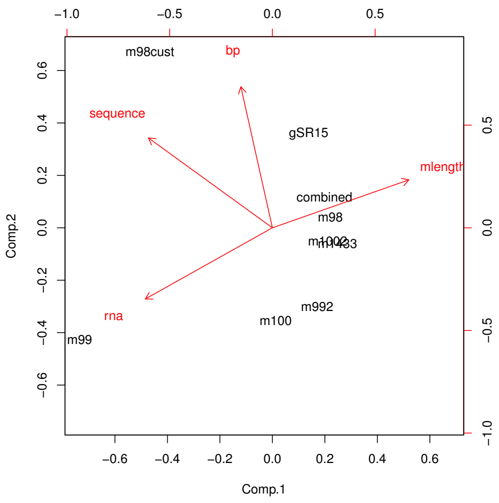

Finally a summary of all sequence data.

```
stats[, -1]

               bp sequence mlength   sd mgc feature functional rna
m98      24868199    15489    1605 1765  45    5536       3598  21
m98cust  45010375    39946    1126 1505  42    5885       3704  60
m992     14370962    11767    1221  921  46    3632       2476   7
m1433    21934277    13557    1617 1677  46    5038       3285  29
m1002    21633746    14651    1476 1133  45    4476       3011   9
m99      18237292    28498     639  504  49    5878       3807 229
m100     17540863    12579    1394 1042  46    4249       2885 121
gSR15    34662469    21280    1628 2907  44    4107       2578   9
combined 27842190    16662    1670 1908  45    5592       3663  34
```

5 Applied annotations, subsytem predictions, and taxonomic distribution

Like the title implies, identified and predicted annotations and protein features are mapped to public sequence libraries.

Reshape data, transform columns into rows.

↰ This annotation has been done with MG-RAST. Visit here for a description of their fast annoation server

```
predicted <- read.xls("./data/libraries.xlsx", sheet = 3)
predicted <- gather(predicted, "ko", "count", 3:8, na.rm = TRUE)
summary(predicted)

       lib      chart         ko                 count
 combined: 6   ko:54   Length:54          Min.   :  31
 gSR15   : 6           Class :character   1st Qu.: 132
 m100    : 6           Mode  :character   Median : 272
 m1002   : 6                              Mean   : 361
 m1433   : 6                              3rd Qu.: 589
 m98     : 6                              Max.   :1471
 (Other) :18
```

Plot difference in identified protein features between libraries.

```
ggplot(predicted,
```

```
       aes(x = lib,
           y = count,
           group = factor(ko))) +
    geom_line(size = .2) +
        geom_point(aes(shape = factor(ko),
                       size = 1.5)) +
                       theme_bw() +
                           geom_text(aes(x = lib,
                                         y = count,
                                         ymax = count,
                                         label = count,
                                         size = 1.5,
                                         hjust = ifelse(sign(count)>1, .5, 0)),
                                     position = position_dodge(width = 1)) +
                           labs(x = "QPX libraries",
                                y = "Counts")
```

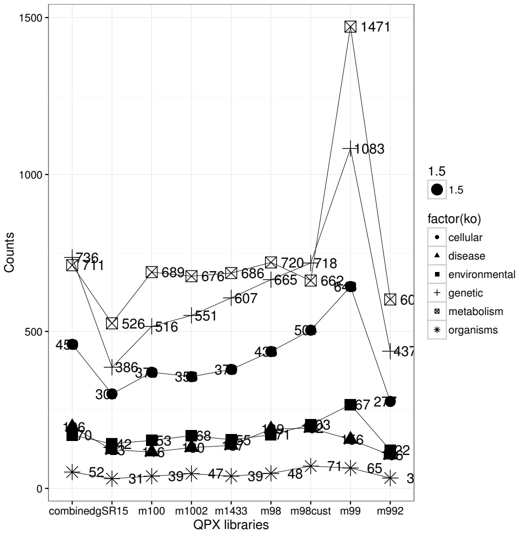

In this next snippet subsystems are discussed. Functional coupling and chromosomal clusters are shown for *clustering-based subsytems* among other subsytems.

```
predicted <- read.xls("./data/libraries.xlsx", sheet = 4)
predicted <- gather(predicted, "subsystems", "count", 3:8, na.rm= TRUE)
ggplot(predicted,
       aes(x = lib,
           y = count,
           group = factor(subsystems))) +
    geom_line(size = .2) +
        geom_point(aes(shape = factor(subsystems),
                       size = 1.5)) +
                       theme_bw() +
                           geom_text(aes(x = lib,
                                         y = count,
                                         ymax = count,
                                         label = count,
                                         size = 1.5,
                                         hjust = ifelse(sign(count)>1, .5, 0)),
                                     position = position_dodge(width = 1)) +
                           labs(x = "QPX libraries",
                                y = "Counts")
```

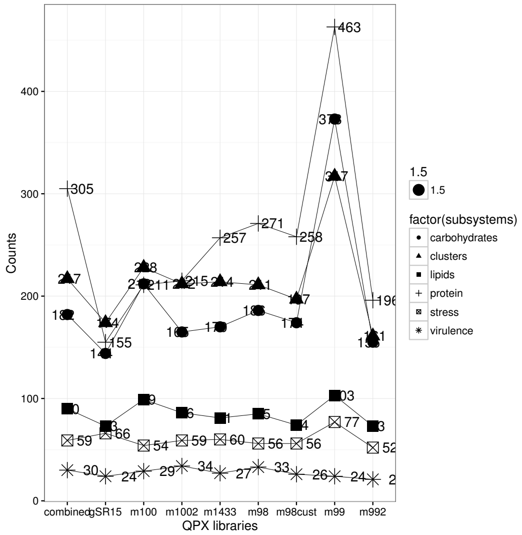

Finally, a taxonomic classification on sequence similarities gives insights on sequence relatedness or sample contamination. Five classes were selected, bacteria, fungi, algae, parasite, and bivalvia.

```
predicted <- read.xls("./data/libraries.xlsx", sheet = 5)
predicted <- gather(predicted, "class", "count", c(3,5:9), na.rm = TRUE)
ggplot(predicted,
       aes(x = lib,
           y = count,
           group = factor(class))) +
    geom_line(size = .2) +
        geom_point(aes(shape = factor(class),
                       size = 1.5)) +
                       theme_bw() +
                           geom_text(aes(x = lib,
                                         y = count,
                                         ymax = count,
                                         label = count,
                                         size = 1.5,
                                         hjust = ifelse(sign(count)>1, .5, 0)),
                                     position = position_dodge(width = 1)) +
                           labs(x = "QPX libraries",
                                y = "Counts")
```

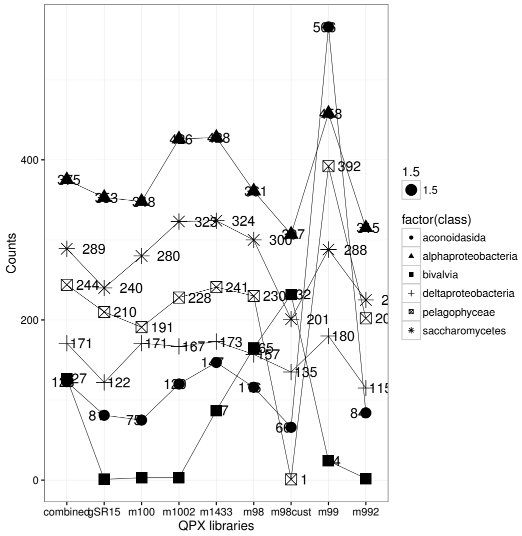

6 Shared SNPs between libraries

Shared SNPs between libraries mapped to SR genome v15. The first column shows the count of shared SNPs. The next 3 columns shows the name of the QPX library (mmetsp00 98, 992,

↰ A higher resolution summary of the 4 last plots can be found in Supplemental Information

1002, 1433). The libraries situated on a same row share the same SNPs.

```
shared.snps <- read.table("./data/shared.snps.txt", fill =TRUE)
shared.snps
```

```
                  X132 X1433.0.9.. X992.1.9..
200           98(1.2%)   992(2.9%)
314         1002(3.2%)  1433(2.1%)
328         1002(3.4%)  1433(2.1%)  992(4.7%)
587         1002(6.0%)    98(3.5%)  992(8.4%)
589         1433(3.9%)    98(3.5%)  992(8.5%)
632         1002(6.5%)    98(3.8%)
655          992(9.4%)
825         1002(8.5%)  992(11.8%)
1679       1002(17.3%)
1702       1002(17.5%) 1433(11.1%)  98(10.2%)
2577       1433(16.9%)
3394         98(20.3%)
3649       1002(37.6%) 1433(23.9%)  98(21.8%)
992(52.4%)
5976       1433(39.1%)   98(35.7%)
```

Shared indels between libraries mapped to SR genome v15.

↰ A venn diagram of the shared SNPs can be found in the Manuscript

```
shared.indels <- read.table("./data/shared.indels.txt", fill = TRUE)
shared.indels
```

```
                    X8   X98.0.7.. X992.2.0..
12          1433(1.1%)   992(3.1%)
14          1002(2.7%)  1433(1.3%)  992(3.6%)
15          1002(2.9%)  1433(1.4%)
31          1002(5.9%)    98(2.7%)
40          1433(3.7%)    98(3.4%) 992(10.2%)
41          1002(7.8%)    98(3.5%) 992(10.5%)
62         1002(11.8%)  992(15.9%)
68          992(17.4%)
78         1002(14.8%)  1433(7.2%)   98(6.7%)
139        1002(26.4%)
146        1002(27.8%) 1433(13.5%)  98(12.5%)
992(37.3%)
267        1433(24.7%)
316          98(27.1%)
507        1433(47.0%)   98(43.4%)
```

6.1 Component analysis and sequence closeness from MG-RAST annotation

Import annotated data.

```
closeness <- read.csv("./data/pca.csv", sep = "\t")
```

```r
summary(closeness)
```

```
      metagenome
 mmetsp1002:1141
 mmetsp1433:1278
 mmetsp98  :1279
 mmetsp992 :1088
 QPX_v15   :1030


                                            level.1
 Carbohydrates                                  : 848
 Amino Acids and Derivatives                    : 785
 Protein Metabolism                             : 769
 Clustering-based subsystems                    : 559
 Miscellaneous                                  : 552
 Cofactors, Vitamins, Prosthetic Groups, Pigments: 462
 (Other)                                        :1841
                            level.2
 0                               : 750
 Plant-Prokaryote DOE project    : 506
 Protein biosynthesis            : 499
 RNA processing and modification: 346
 Central carbohydrate metabolism: 326
 Folate and pterines             : 278
 (Other)                         :3111
                                 level.3
 YgfZ                                 : 120
 Ribosome LSU eukaryotic and archaeal: 101
 Proteasome eukaryotic                :  91
 Ribosome SSU eukaryotic and archaeal:  91
 Serine-glyoxylate cycle              :  88
 tRNA modification Bacteria           :  76
 (Other)                              :5249
                                                         function.
 GTP cyclohydrolase I (EC 3.5.4.16) type 1               :  70
 Acetyl-CoA acetyltransferase (EC 2.3.1.9)               :  55
 Serine hydroxymethyltransferase (EC 2.1.2.1)            :  50
 Cysteine desulfurase (EC 2.8.1.7)                       :  48
 3-ketoacyl-CoA thiolase (EC 2.3.1.16)                   :  40
 Branched-chain amino acid aminotransferase (EC 2.6.1.42):  40
 (Other)                                                 :5513
   abundance      avg.eValue    avg...ident     avg.align.len
 1      :3943   Min.   :-269   Min.   :-183.0   Min.   : 24
 2      :1140   1st Qu.: -57   1st Qu.:  63.2   1st Qu.: 61
 3      : 280   Median : -29   Median :  66.2   Median : 92
 4      : 155   Mean   : -41   Mean   :  65.7   Mean   :118
 5      :  69   3rd Qu.: -15   3rd Qu.:  70.2   3rd Qu.:156
 6      :  68   Max.   :   3   Max.   :  95.6   Max.   :544
 (Other): 161
    X..hits          X
 Min.   :  1   Min.   :1
 1st Qu.:  1   1st Qu.:1
 Median :  1   Median :1
 Mean   :  4   Mean   :1
 3rd Qu.:  2   3rd Qu.:2
 Max.   :457   Max.   :3
               NA's   :5727
```

```r
closeness <- closeness[, c(1, 7:10)]
```

Principal component analysis on 5 libraries, 4 strains and the genome (v15), using an *identity score* for annotating a sequence and an *alignment length score* for similarities with estimated functional features, an *e-value score* for estimated functional similarities, and the *number of hits*, ie., the number of times a function is identified in a library.

```r
rownames(closeness) <- paste(closeness[, 1], 1:nrow(closeness), sep = ".")
x=closeness[, -1]
head(x)

             avg.eValue avg...ident avg.align.len X..hits
mmetsp1433.1        -57        74.7           142       1
QPX_v15.2           -57        74.7           142       1
mmetsp98.3          -57        74.7           142       1
mmetsp1002.4        -57        74.7           142       1
mmetsp1433.5        -60        64.7           173       1
QPX_v15.6           -60        64.7           173       1

## standardization (columns)
results <- decostand(x, method = "range")
head(results)

             avg.eValue avg...ident avg.align.len X..hits
mmetsp1433.1      0.779       0.925         0.227       0
QPX_v15.2         0.779       0.925         0.227       0
mmetsp98.3        0.779       0.925         0.227       0
mmetsp1002.4      0.779       0.925         0.227       0
mmetsp1433.5      0.768       0.889         0.287       0
QPX_v15.6         0.768       0.889         0.287       0

p = princomp(~ avg...ident + avg.align.len
   , data= results)
summary(p)

Importance of components:
                       Comp.1 Comp.2
Standard deviation      0.156 0.0605
Proportion of Variance  0.869 0.1309
Cumulative Proportion   0.869 1.0000

#plot(p, type = "l")
#biplot(p, cex = .4)
```

Clustering and visualization of all sequences without applying any filters.

```r
ggbiplot(p, obs.scale = 1,
         var.scale = 1,
         groups = closeness$metagenome,
         ellipse = TRUE,
         circle = FALSE) +
        geom_point(aes(size = closeness$X..hits)) +
        theme_bw() +
            theme(legend.direction = 'horizontal',
                  legend.position = 'top')
```

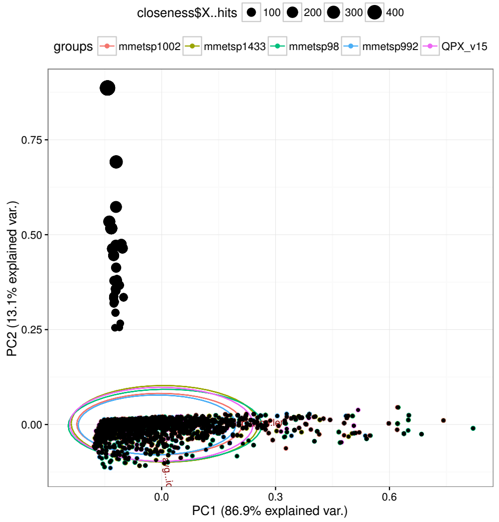

Build a custom PCA function for repetitive iterations.

```
customBiplot <- function(data, method){
x=data[, -1]
results <- decostand(x, method = method)
p = princomp(~ results[, 2] + results[, 3]
   , data= results)
ggbiplot(p, obs.scale = 1,
         var.scale = 1,
         groups = data$metagenome,
         ellipse = TRUE,
         circle = FALSE) +
        theme_bw() +
            theme(legend.direction = 'horizontal',
                  legend.position = 'top')
}
```

Filter sequences depending on their alignment length and the abundance of an identified function (therotically a protein).

```
closenessX <- filter(closeness, avg.align.len < 50, X..hits > 2)
dim(closenessX)[1]

[1] 57

customBiplot(closenessX, method = "range")
```

↰ results[,2] = identity and results[,3] = alignment length

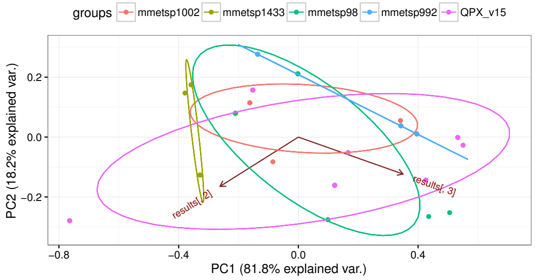

Filter by selecting higher alignment scores only.

```
closenessX <- filter(closeness, avg.align.len < 60, X..hits > 2)
dim(closenessX)[1]
```

```
[1] 117
```

```
customBiplot(closenessX, method = "range")
```

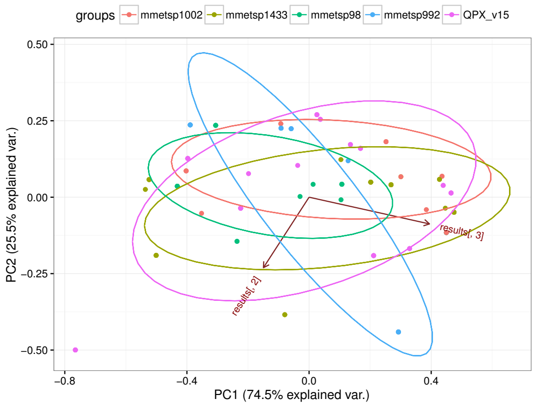

Select even higher alignment similarities.

```
closenessX <- filter(closeness, avg.align.len < 100, X..hits > 4)
dim(closenessX)[1]
```

```
[1] 199
```

```
customBiplot(closenessX, method = "range")
```

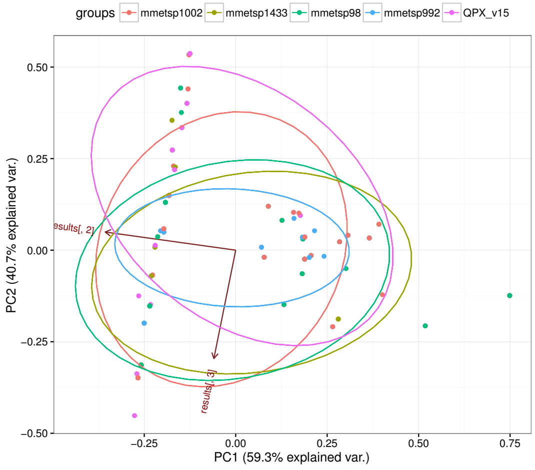

Select on the criteria of e-Value and abundance of a functional sequence.

```
closenessX <- filter(closeness, avg.eValue < -40, X..hits > 2)
dim(closenessX)[1]
```

```
[1] 152
```

```
customBiplot(closenessX, method = "range")
```

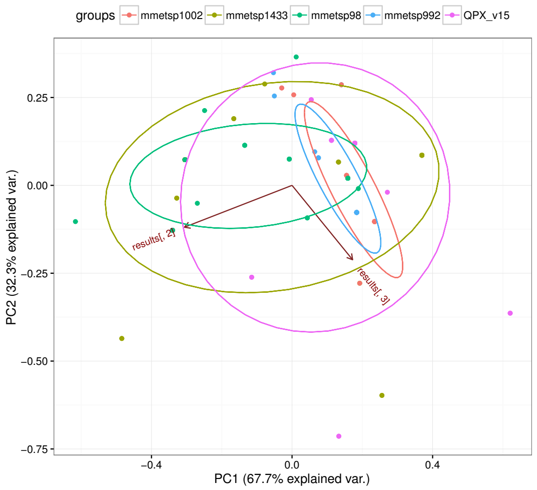

Select on the criteria of e-Value and abundance of a functional sequence.

```
closenessX <- filter(closeness, avg.eValue < -40, X..hits > 3)
dim(closenessX)[1]

[1] 126

customBiplot(closenessX, method = "range")
```

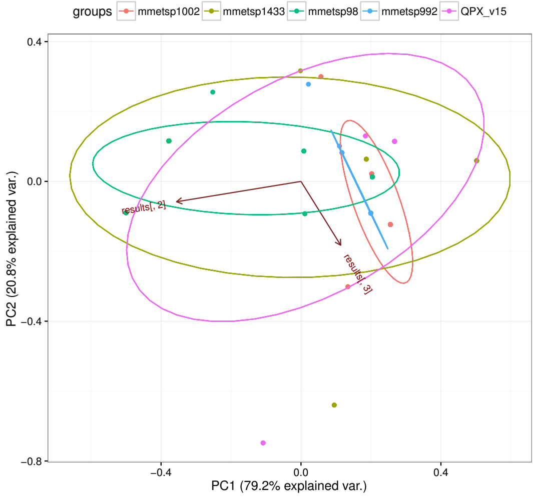

Select on the criteria of e-Value and abundance of a functional sequence.

```
closenessX <- filter(closeness, avg.eValue < -50, X..hits > 2)
dim(closenessX)[1]

[1] 109

customBiplot(closenessX, method = "range")
```

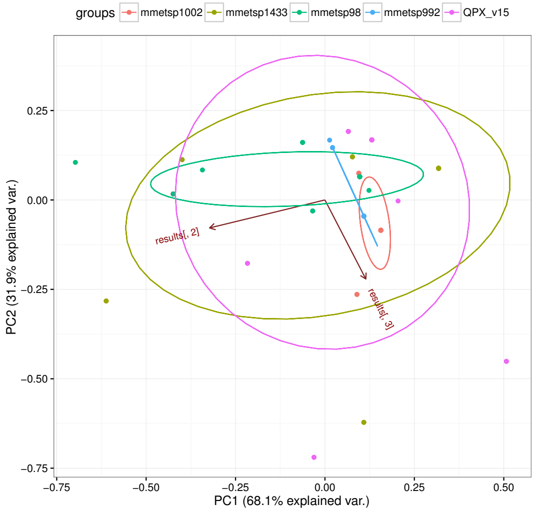

Select on the criteria of alignment length. Since SNP aggregation tests are the next step in this analysis, the length of a correct alignment is technically helpful in differentiating SNP position. And abundance will be more than 2 to increase probabilities of correct functional annotation.

↰ This final biplot represent a successful unsupervised clustering of QPX strains. Differences between strains is reflected by the nature of the assembled contigs. But also to the nature of each base pair in these sequences. Top hit contigs N=100

```
closenessX <- filter(closeness, avg.align.len > 200, X..hits >= 2)
dim(closenessX)[1]

[1] 100

customBiplot(closenessX, method = "range")
```

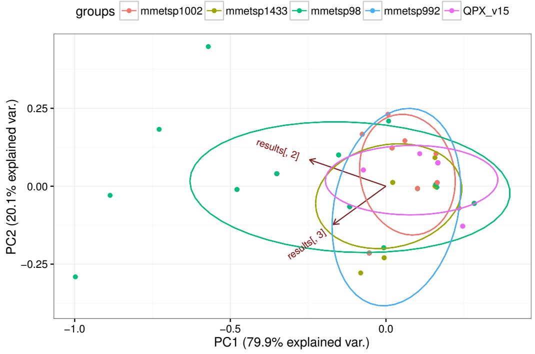

7 Aggregation analysis of SNPS

MMETSP libraries are already been annotated. How many contigs, peptide and cds elements are indexed?

↰ Only mmetsp 98, 992, 1002, and 1433 are used in the remaining tests

```
contigs <- read.xls("./data/annot.stats.xlsx", sheet = 1)
```

```
contigs <- gather(contigs, "elements", "counts", 2:4)
ggplot(contigs,
       aes(x = factor(library),
           y = counts,
           fill = factor(elements))) +
    geom_bar(stat = "identity",
             position = "dodge") +
        theme_bw() +
            theme(legend.direction = 'horizontal',
                  legend.position = 'top') +
                coord_flip() +
                    geom_text(aes(x = factor(library),
                                  y = counts,
                                  ymax = counts,
                                  label = counts,
                                  hjusts = ifelse(sign(counts)> 0,1,0)),
                              position = position_dodge(width = 1)) +
                    labs(x = "Counts",
                         y = "QPX libraries")
```

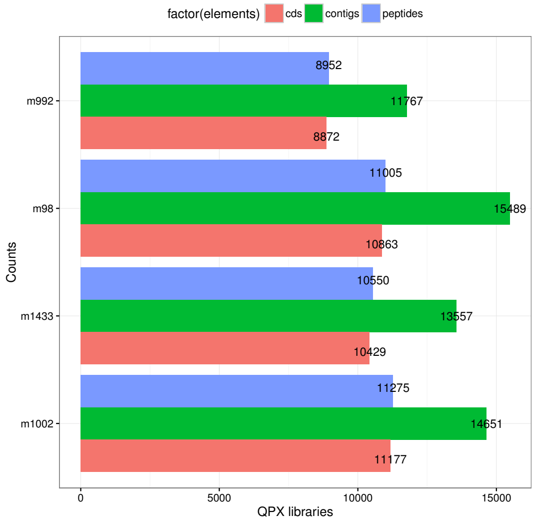

7.1 Preferential substitution

Preferential substitution of nucleotides. It should be noted that *mmetsp0098* and *mmetsp1433* are both bigger in library size than the others. Therefore comparison of SNPs should be done for each library separately. However there is a resemblance in substitution between libraries since the pattern is quite similar for all nucleotides.

```
prefs <- read.table("./data/all.stats.txt")
prefs$V3 <- c(rep("m1002", 12),
              rep("m98", 12),
              rep("m992", 12),
              rep("m1433", 12))
ggplot(prefs,
       aes(x = factor(V1),
           y = V2,
           fill = factor(V3))) +
    geom_bar(stat = "identity",
             position = "dodge") +
        theme_bw() +
    labs(x = "Nucleotide substitution within called SNPs",
         y = "Counts")
```

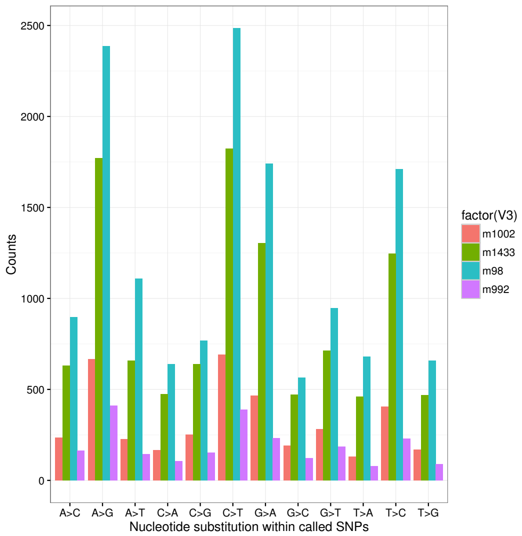

After hard filtering SNPs to the minimum from all 4 libraries, *DISCARD*-labelled SNPs were removed. The remaining were imported into data frames with the following columns.

1. CHROM: number of contig
2. POS: SNP position on that contig
3. ALT: alternative SNP to the reference
4. AD: allelic depth for the reference and ALT alleles
5. DP: approximate read depth
6. GQ: genotype quality
7. PL: normalized phred scaled likelihoods

The structure of the data frame is similar to the *iris* data.

↰ With low number of sample it is impossible to create a f(SNP)=strain machine learning framework. To make a binary table of SNPs at least 100 samples must be used.

```
x <- c('m98', 'm1433', 'm1002', 'm992')
y <- c(16729, 15267, 9716, 6965)
dat <- data.frame(lib = x, SNPs = y)
ggplot(dat,
       aes(x = lib,
           y = SNPs)) +
    geom_bar(stat = "identity") +
        theme_bw() +
            coord_flip() +
                geom_text(aes(x = lib,
                              y = SNPs,
                              ymax = SNPs,
                              label = SNPs,
                              size = 5,
                              col = "white",
                              hjust = 2)) +
                labs(x = "Number of called SNPs with GATK",
                     y = "QPX libraries")
```

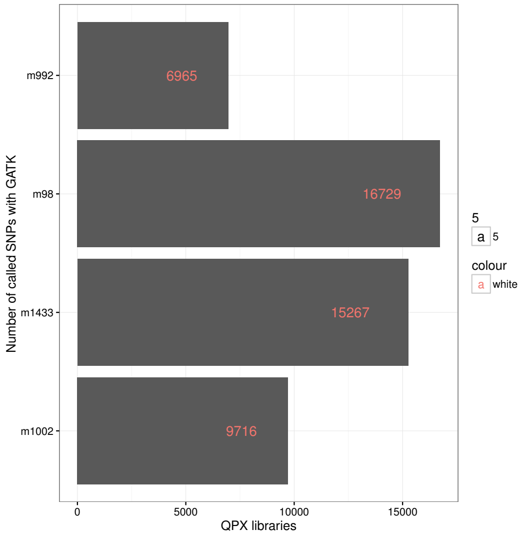

Import SNP data: data manipulation process of removing NAs and getting the same number of SNPs across all samples.

```
m98 <- read.table("./data/m98.ml.txt", fill = NA)
m1433 <- read.table("./data/m1433.ml.txt", fill = NA)
m992 <- read.table("./data/m992.ml.txt", fill = NA)
m1002 <- read.table("./data/m1002.ml.txt", fill = NA)

colnames(m98) <- c('contigs', 'pos', 'ad1', 'ad2',
                   'dp', 'gq', 'pl1', 'pl2', 'pl3', 'lib')
colnames(m1433) <- c('contigs', 'pos', 'ad1', 'ad2',
                   'dp', 'gq', 'pl1', 'pl2', 'pl3', 'lib')
colnames(m992) <- c('contigs', 'pos', 'ad1', 'ad2',
                   'dp', 'gq', 'pl1', 'pl2', 'pl3', 'lib')
colnames(m1002) <- c('contigs', 'pos', 'ad1', 'ad2',
                   'dp', 'gq', 'pl1', 'pl2', 'pl3', 'lib')

m98 <- m98[complete.cases(m98), ]
m1433 <- m1433[complete.cases(m1433), ]
m992 <- m992[complete.cases(m992), ]
m1002 <- m1002[complete.cases(m1002), ]

m98 <- m98[! m98$lib != 'm98', ]
m1433 <- m1433[! m1433$lib != 'm1433', ]
m992 <- m992[! m992$lib != 'm992', ]
m1002 <- m1002[! m1002$lib != 'm1002', ]

m1433$pl3 <- as.numeric(m1433$pl3)

m98$lib <- factor(m98$lib, "m98")
m992$lib <- factor(m992$lib, "m992")
m1433$lib <- factor(m1433$lib, "m1433")
m1002$lib <- factor(m1002$lib, "m1002")

index <- min(dim(m98)[1], dim(m1433)[1],
             dim(m992)[1], dim(m1002)[1])
set.seed(123)
mall <- rbind(m98[sample(nrow(m98), index), ],
              m1433[sample(nrow(m1433), index), ],
              m992[sample(nrow(m992), index), ],
              m1002[sample(nrow(m1002), index), ])
dim(mall)

[1] 27844    10
```

7.2 Distribution of SNPs in the QPX libraries

Regressing the genome quality of SNPs on the position of the SNPs inside a contig. This shows that SNPs are concentrated in the first 10 Kb.

```r
head(mall)

      contigs pos ad1 ad2 dp gq pl1 pl2 pl3 lib
4825     5776 244   5  18 23 99 786   0 150 m98
13214   14964  96   6   6 12 99 234   0 234 m98
6857     7810 176   3  11 14 68 392   0  68 m98
14801   17502  56   9   7 16 99 230   0 337 m98
15760   19016  80   0   7  7 21 315  21   0 m98
763       884 810  17  29 46 99 986   0 516 m98

with(mall, plot(pos, gq, cex = .5))
```

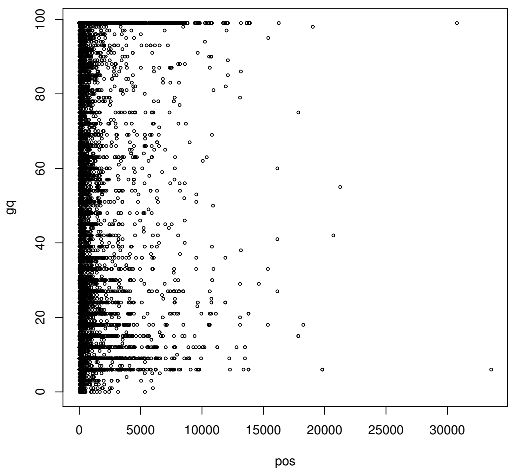

Regression of contigs and the read depth for each SNP in those contigs. When using libraries mapped to the combined assembly (as a reference transcriptome, not showing here) the plot shows that the depth of coverage cat split the SNPs inside the QPX contigs into 2 separate subsets. However the regression is constant when using the genome of SR v15 as a reference for mapping the libraries (as shown below).

```r
with(mall, plot(contigs, dp, cex = .5))
submall <- filter(mall, dp > 50, pos <= 10000)
```

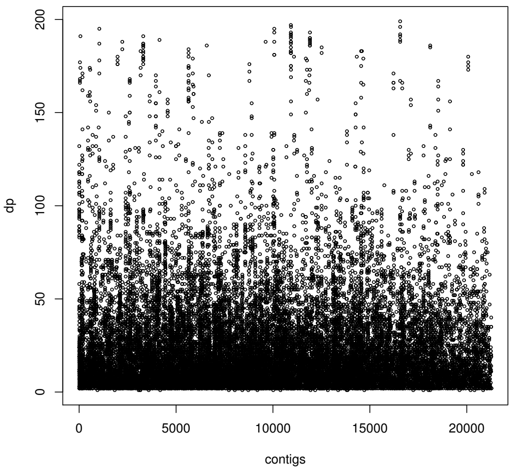

This plot shows that 11.41 % of the SNPs have a depth over 50 for the first 10 Kb QPX contigs size.

```r
with(mall, plot(pos, dp, cex = .5))
## percentage of SNPs with read depth higher than 35
(nrow(submall)/nrow(mall))*100
```

```
[1] 11.4
```

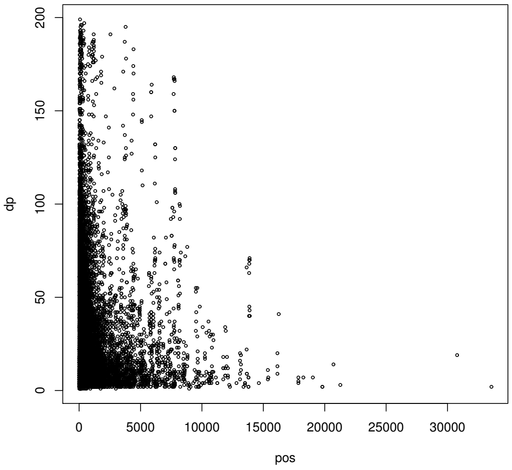

Plotting only SNPs with DP > 50 and in contigs which length <= 10 Kb, and regressing toward a phred-scaled adjusted likelihood for each variant or genotype likelihood.

```r
with(submall, plot(pos, pl1, cex = .5))
summary(submall$lib)
```

```
  m98 m1433  m992 m1002
 1256  1127   417   377
```

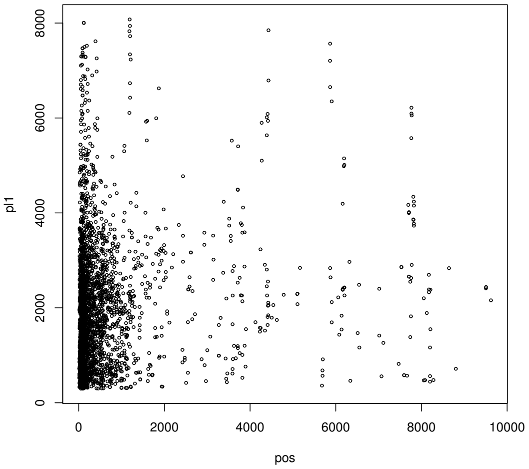

Linear regression between position of the SNP and the normalized phred scaled likelihood, which on its own is an accuracy determination score. Phred likelihoods (PL) are computed for the REF/REF, REF/ALT, and ALT/ALT variants. To convert a PL to a raw likelihood L:

$$P(L|AA) = 10^{-P_{AA}/10} \tag{3}$$

These probabilities are adjusted with phred scores. They determine the probability of a base observed given a reference genotype, an heterozygous genotype or a non-reference genotype respectively (pl1, pl2, and pl3).
Accordingly, REF/REF (pl1) is significant. Meaning the genotype we have is homozygous for the reference nucleotide (not the variant), but if a variant exists, thus it represents a rare mutation (*reference needed*). Therefore, the raw likelihoods must be calculated with the equation above for the picked variants and the genotype with *P=1* is the most significant genotype at that nucleotide.

```
fit <- lm(pos~pl1, data = submall)
summary(fit)


Call:
lm(formula = pos ~ pl1, data = submall)

Residuals:
   Min     1Q Median     3Q    Max 
 -1353   -484   -344    -97   8996 

Coefficients:
            Estimate Std. Error t value Pr(>|t|)    
(Intercept) 315.2290    39.9153    7.90  3.9e-15 ***
pl1           0.1442     0.0171    8.42  < 2e-16 ***
---
Signif. codes:  0 '***' 0.001 '**' 0.01 '*' 0.05 '.' 0.1 ' ' 1

Residual standard error: 1280 on 3175 degrees of freedom
Multiple R-squared:  0.0219,Adjusted R-squared:  0.0216 
F-statistic:   71 on 1 and 3175 DF,  p-value: <2e-16
```

Lets get the variants with the highest probability that a genotype has been identified. *PL=1* determines the genotype, either homozygous for REF (pl1) or ALT (pl3) or heterozygous REF/ALT (pl2).

```
submall[, 7:9] <- apply(submall[, 7:9], 2, function(x) 10^(-x/1000))
head(submall)

  contigs pos ad1 ad2  dp gq     pl1 pl2        pl3 lib
1    6470 215  43  21  64 99 0.18113   1 0.01717908 m98
2   20921  54  88  21 109 99 0.22751   1 0.00000196 m98
3   13280  47  78  19  97 99 0.27542   1 0.00054954 m98
4   10194 378   5  49  54 63 0.01064   1 0.86496792 m98
5   19812  80  55  41  96 99 0.04027   1 0.00693426 m98
6    4446  65  15  65  80 99 0.00514   1 0.49545019 m98
```

Lets extract all heterozygous alleles with at least 90 % confidence.

```
heteromall <- filter(submall, pl2 >= .9)
```

The original total number of SNPs was 3177 among which the total number of variants with an heterozygous genotype is 2554.

↰ A summary of the plots in this subsection can be found in the Supplemental Information

```
dat <- as.data.frame(summary(heteromall$lib))
ggplot(dat,
       aes(x = rownames(dat),
           y = dat[, 1])) +
    theme_bw() +
        geom_bar(stat = "identity") +
            coord_flip() +
                geom_text(aes(x = rownames(dat),
                              y = dat[, 1],
                              ymax = dat[, 1],
                              size = 5,
                              label = dat[, 1],
                              col = "white",
                              hjust = 2)) +
                labs(x = "Number of heterozygous SNPs",
                     y = "QPX librairies")
```

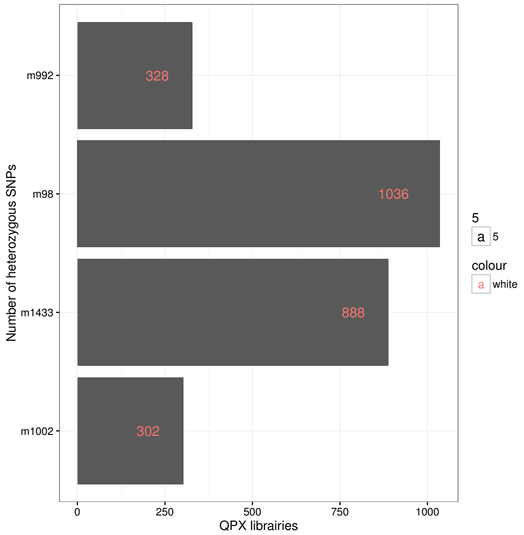

Now lets get the homozygous variants with genotype ALT/ALT with 90 %.

```
altmall <- filter(submall, pl3 >= .9)
```

The total number of variants ALT/ALT is 771. Interesting thing is that using the combined assembly as a reference (not showing here), m1433 had also the highest number of homozygous alleles while m98 had half the number shown below.

↰ The same analysis was done twice with genome reference and the combined assembly as reference

```
dat <- as.data.frame(summary(altmall$lib))
ggplot(dat,
       aes(x = rownames(dat),
           y = dat[, 1])) +
    theme_bw() +
        coord_flip() +
            geom_bar(stat = "identity") +
                geom_text(aes(x = rownames(dat),
                              y = dat[, 1],
                              ymax = dat[, 1],
                              label = dat[, 1],
                              size = 5,
                              color = "white",
                              hjust = 2)) +
                labs(x = "Number of homozygous SNPs",
                     y = "QPX librairies")
```

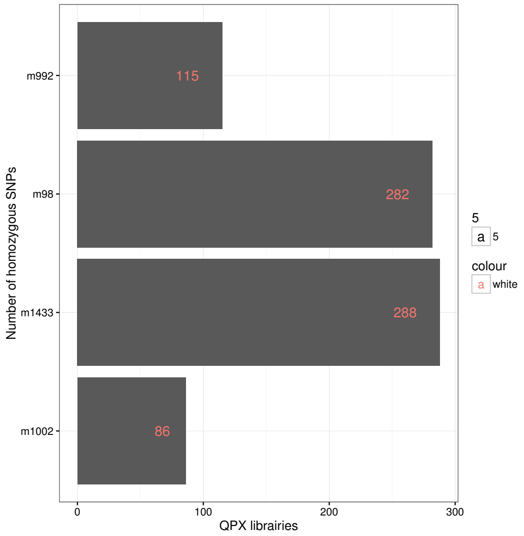

8 Protein domain annotation

Get the number of protein domains that can be predicted from the MMETSP strains. First, assembled contigs must be translated into peptides. HMMER3.2b was used for annotation. Hidden

Markov Models were generated on Pfam database. The table below lists old and new annotations against old and new Pfam v26 and v28 libraries. (> 2 years interval between versions).

```
pfam <- read.xls("./data/pfam.xlsx", sheet = 1)
pfam

          domain pfam  a98 s98 a992 s992 a1002 s1002 a1433 s1433
1      virulence  655 5098 313 3075  261  4606   291  4794   308
2    temperature  251 2484 168 1680  141  2283   164  2277   161
3       salinity   22  163  13   91    9   123    10   137    12
4 salt tolerance   79 2231  70 1422   64  2097    66  2078    66
5      virulence  655 5306 331 3185  275  4763   302  4973   326
6    temperature  251 2704 179 1771  145  2436   170  2478   170
7       salinity   22  161  12   97   10   128    10   138    10
8 salt tolerance   79 2267  73 1451   68  2108    67  2138    69
  annot
1   old
2   old
3   old
4   old
5   new
6   new
7   new
8   new
```

Number of domains found in Pfam v28 for :

- Virulence
- Temperature
- Salinity
- Salt tolerance

```
ggplot(pfam[1:4, ],
       aes(x = domain,
           y = pfam)) +
    coord_flip() +
        theme_bw() +
            geom_bar(stat = "identity") +
                geom_text(aes(x = domain,
                              y = pfam,
                              ymax = pfam,
                              label = pfam,
                              size = 5,
                              color = "white",
                              hjust = 2)) +
                labs(x = "Number of domains in each sub library of Pfam",
                     y = "Biological relevance of each Pfam subsets")
```

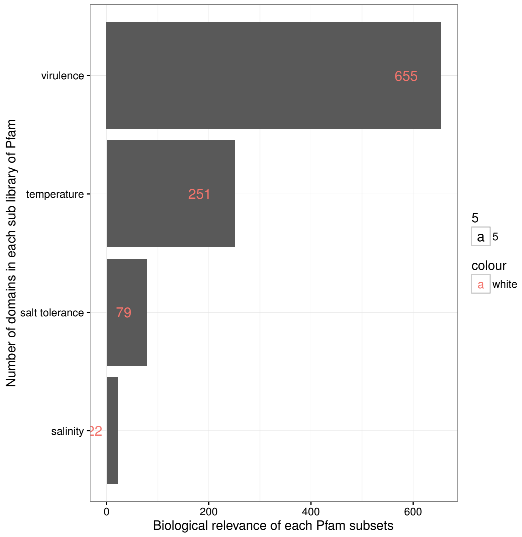

The number of domains that can be estimated from 4 QPX strains. For example if 2 totally different contigs are aligned to one same domain the counter is incremented by 2.

```
allpfam <- select(pfam, contains("a"))
allpfam <- filter(allpfam, annot == "new")
allpfam

          domain pfam  a98 a992 a1002 a1433 annot
1      virulence  655 5306 3185  4763  4973   new
2    temperature  251 2704 1771  2436  2478   new
3       salinity   22  161   97   128   138   new
4 salt tolerance   79 2267 1451  2108  2138   new

allpfam <- gather(allpfam[, -2], "lib", "count", 2:5)
ggplot(allpfam,
       aes(x = lib,
           y = count,
           fill = domain)) +
    theme_bw() +
        coord_flip() +
            geom_bar(stat = "identity",
                     position = "dodge") +
                geom_text(aes(x = lib,
                              y = count,
                              ymax = count,
                              label = count,
                              size = 5,
                              hjust = 1),
                               position = position_dodge(width = 1)) +
                labs(x = "Count of identified peptides in Pfam",
                     y = "QPX libraries")
```

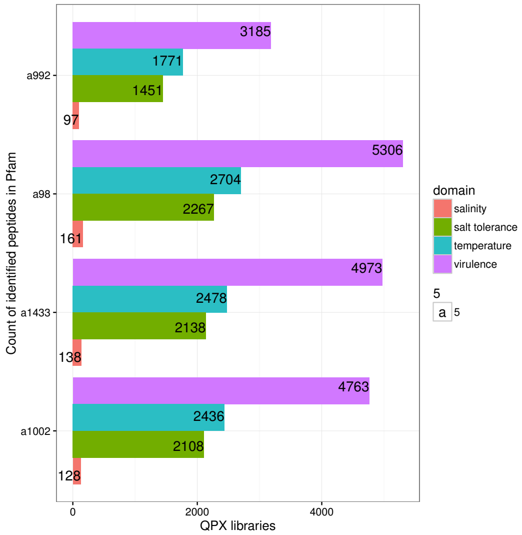

The *unique* number of domains identified from the alignment. For example, if 2 totally different contigs are aligned to one domain the counter is incremented by 1.

```
singlepfam <- select(pfam, contains("s"))
singlepfam <- cbind(singlepfam, X = pfam$domain, Y = pfam$annot)
singlepfam <- filter(singlepfam, Y == "new")
singlepfam <- gather(singlepfam, "lib", "count", 1:4)
ggplot(singlepfam,
       aes(x = X,
           y = count,
           fill = lib)) +
    theme_bw() +
        coord_flip() +
            geom_bar(stat = "identity",
                     position = "dodge") +
                geom_text(aes(x = X,
                              y = count,
                              ymax = count,
                              label = count,
                              size = 5,
                              hjust = 1),
                         position = position_dodge(width = 1)) +
                labs(x = "Count of Pfam domains aligned to QPX peptides",
                     y = "Pfam domain subsets")
```

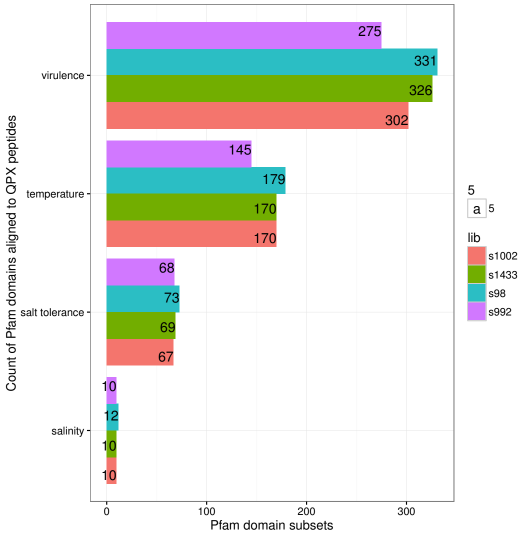

Difference in domain-peptide alignments between old and new pfam databases. Numbers on the right belong to the new Pfam library. Numbers on the left belong to the old Pfam library.

```
newpfam <- select(pfam, contains("s"))
newpfam <- cbind(newpfam, annot = pfam$annot, domain = pfam$domain)
newpfam <- gather(newpfam, "lib", "count", 1:4)
ggplot(newpfam,
       aes(x = domain,
           y = count,
           fill = annot,
           group = lib)) +
    theme_bw() +
        coord_flip() +
            geom_bar(stat = "identity",
                     position = "dodge") +
                geom_text(aes(x = domain,
                              y = count,
                              ymax = count,
                              label = count,
                              size = 5,
                              hjust = 1),
                          position = position_dodge(width = 1)) +
                labs(x = "Count of Pfam domains old (left) and new (right) aligned to QPX",
                     y = "Pfam domain subsets")
```

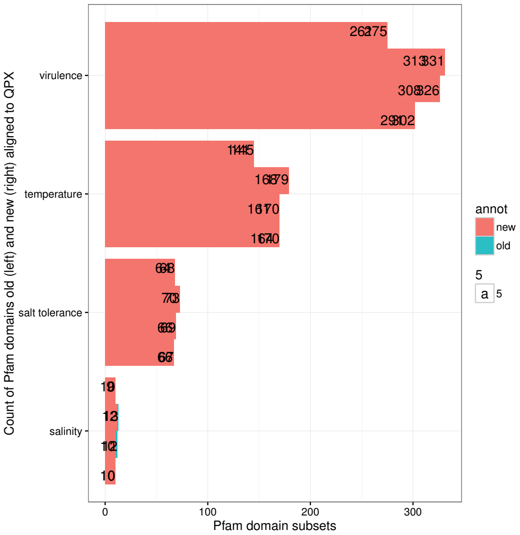

Get the number of peptides that match a significant e-value domain.

↰ The new Pfam library will be used for the remaining tests

```
pfam2 <- read.xls("./data/pfam.xlsx", sheet = 2)
pfam2 <- filter(pfam2, annot == "contig")
pfam2 <- gather(pfam2, "evalue", "count", 2:5)
ggplot(pfam2,
       aes(x = pfam,
           y = count,
           fill = evalue,
           group = lib)) +
    theme_bw() +
        geom_bar(stat = "identity",
                 position = "dodge") +
            scale_fill_brewer() +
                coord_flip() +
                    geom_text(aes(x = pfam,
                                  y = count,
                                  ymax = count,
                                  label = count,
                                  size = 4,
                                  hjust = 1),
                              position = position_dodge(width = 1)) +
                    labs(x = "Count of peptides",
                         y = "Pfam domain subsets")
```

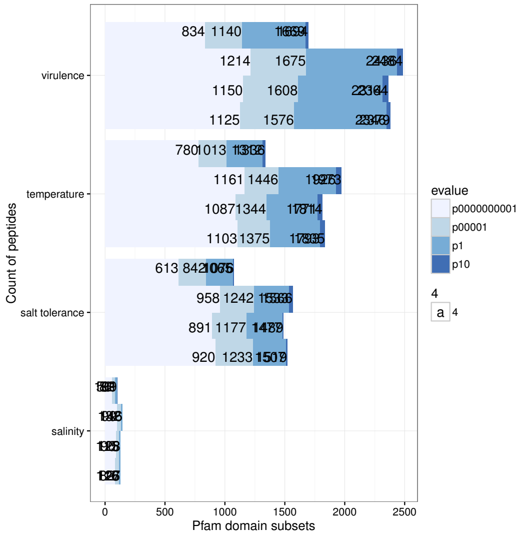

How many *unique* protein domains were found at different evalue significance.

↰ A higher resolution summary of these 2 plots can be found in the Manuscript

```
pfam2 <- read.xls("./data/pfam.xlsx", sheet = 2)
pfam2 <- filter(pfam2, annot == "domain")
pfam2 <- gather(pfam2, "evalue", "count", 2:5)
ggplot(pfam2,
       aes(x = pfam,
           y = count,
           fill = evalue,
           group = lib)) +
    geom_bar(stat = "identity",
             position = "dodge") +
        scale_fill_brewer() +
            coord_flip() +
                theme_bw() +
                    geom_text(aes(x = pfam,
                                  y = count,
                                  ymax = count,
                                  size = 4,
                                  label = count,
                                  hjust = 1),
                              position = position_dodge(width = 1)) +
                    labs(x = "Count of unique domains",
                         y = "Pfam domain subsets")
```

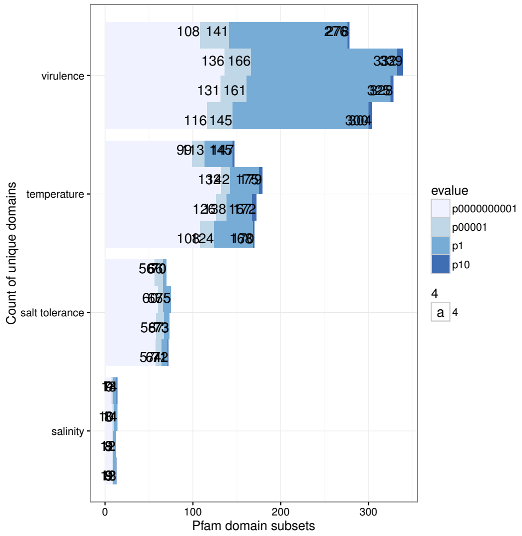

9 Align assembled contigs to Genome (v15)

Here is the overall stats of the BLAT of the 4 strains RNA sequenced contigs against SR. genome v15. The QPX contigs have been annotated with pfam. They are aligned to the reference

genome for SNP localization. Hence we can identify SNP hotspots inside and outside functional domains.

```
blat <- read.table("./data/pfam.stats.genomics.txt", header = T)
x <- c("m98", "m992", "m1002", "m1433")
y <- gl(4, 4, 16, labels = c("virulence", "temperature", "salinity", "salt tolerance"))
blat <- data.frame(blat, lib = c(rep(x, 4)), pfam = y)
ggplot(blat,
       aes(x = pfam,
           y = queryCnt,
           fill = lib)) +
    geom_bar(stat = "identity",
             position = "dodge") +
        theme_bw() +
            coord_flip() +
                scale_fill_brewer() +
                    geom_text(aes(x = pfam,
                                  y = queryCnt,
                                  ymax = queryCnt,
                                  label = queryCnt,
                                  size = 4,
                                  hjust = 1),
                              position = position_dodge(width = 1)) +
                    labs(x = "Number of aligned QPX predicted domains to reference",
                         y = "Pfam subsets")
```

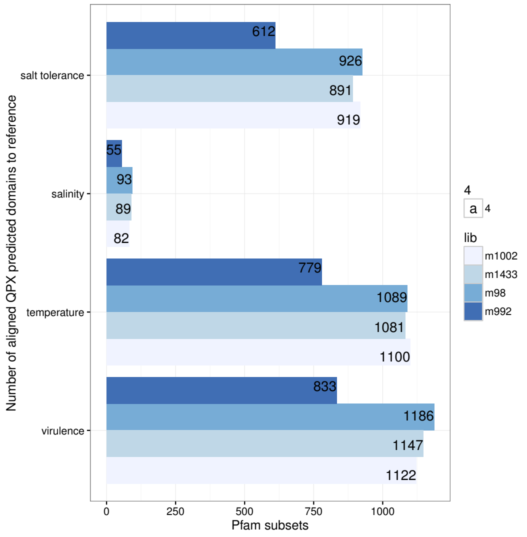

From the table data above the *minimum identity* of all contigs aligned is 0.9. The *mean query* is necessary to choose the number of contigs mapped. Since each contig can be found multiple times in the genome (at different alignment lengths of course) it is best if we choose the best contigs those that have a maximum alignment length (since the PCA analysis has shown previously that a longer alignment is helpful to distinguishing between strains). Contigs must be mapped/aligned once and thus, no duplicate entries should be selected. For this reason choosing an alignment length equal to the half of the mean of the alignment length gives the minimum number of duplicate contigs.

```
ggplot(blat,
```

```
        aes(x = pfam,
            y = meanQSize,
            fill = lib)) +
    coord_flip() +
        theme_bw() +
            geom_bar(stat = "identity",
                     position = "dodge") +
                geom_text(aes(x = pfam,
                              y = meanQSize,
                              ymax = meanQSize,
                              label = meanQSize,
                              size = 4,
                              hjust = 2),
                          position = position_dodge(width = 1)) +
                labs(x = "Mean of the contigs size with predicted Pfam domains",
                     y = "Pfam subsets")
```

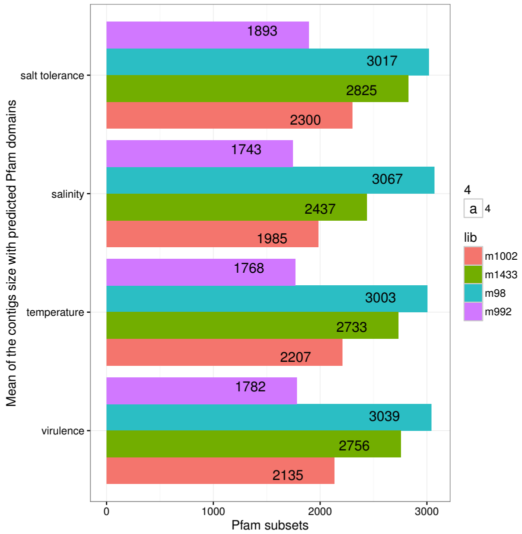

10 Assessing SNP hotspots in 4 QPX strains

QPX contig assemblies by the MMETSP team were used for pfam annotation (with HMMER). SNP calling on the 4 strains used Steve Roberts reference genome v15 (called with GATK). Location of SNPs in the pfam domains was inferred after alignment of the QPX contigs (those that include a prerdicted functional protein domain) on the reference genome (with BLAT). Finally all data were merged in one file grouped by 4 QPX strains (2 from NY, one from each VA and MA) and 3 pfam subset pathways (Virulence, salinity/salt-tolerance, temperature).

```
hotspots.raw <- read.table("./data/hotspots/all.pfam.snp.txt", header = TRUE)
```

↰ This summary file includes SNP location on peptide domains and can be traced back to the 4 strains of QPX and the reference genome. This file can be imported into a MySQL database

What is the correlation between a SNP position and the first reference nucleotide that align to a contig containing domain?

```
ggplot(hotspots.raw,
       aes(x = Position,
           y = Tstart)) +
    theme_bw() +
        geom_point(aes(color = lib,
                       size = Tsize)) +
            facet_wrap( ~ lib, ncol = 2) +
        labs(x = "Position of SNPs in the reference genome",
             y = "Position of the first aligned nucleotide between contig and reference")
```

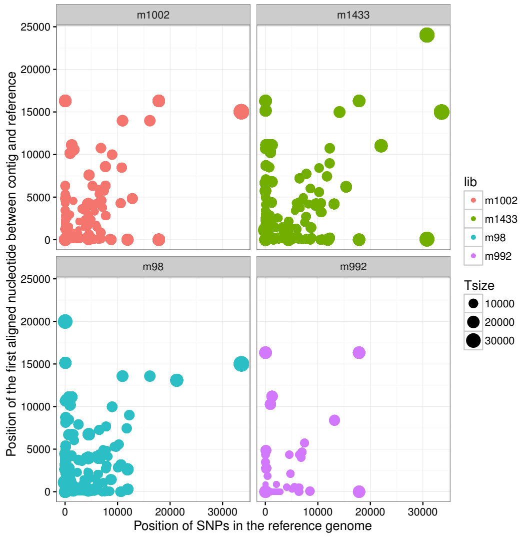

↰ A higher resolution version of this plot can be find in the Manuscript

How many SNPs can be found inside and outside of protein domains, those of which reside in assembled QPX contigs?

```
count <- c(264, 749, 295)
position <- c("Upstream", "Genic", "Downstream")
dat <- data.frame(position, count)
dat$per <- round((dat$count/sum(dat[, 2]))*100, digits = 2)
ggplot(dat,
       aes(x = position,
           y = count)) +
    theme_bw() +
        geom_bar(stat = "identity") +
            coord_flip() +
                geom_text(aes(x = position,
                              y = count,
                              ymax = count,
                              label = per,
                              size = 4,
                              hjust = 2,
                              color = "white")) +
                labs(y = "Number of SNPs (x axis) and % (bar labels)",
                     x = "Position of SNPs relative to contigs aligned to reference")
```

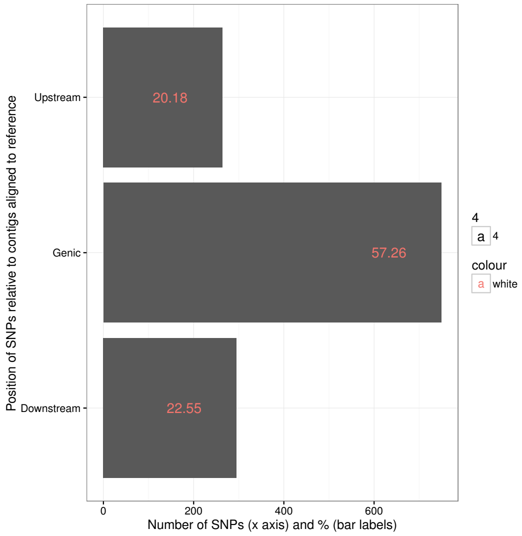

How are SNPs distributed between Pfam subsets? On the chart, the score between parenthesis is the normalized amount of SNPs. It has no units. It is just a score of the normalized counts of SNPs by the number of domains found in each subset. The counts are those of the position of SNPs inside the domains.

```
domain <- c("virulence", "temperature", "salinity", "salt tolerance")
count <- c(467, 306, 64, 347)
dat <- data.frame(domain, count)
dat$norm <- round(dat$count/pfam[1:4, 2], digits = 2)
ggplot(dat,
       aes(x = domain,
           y = count)) +
    theme_bw() +
        coord_flip() +
            geom_bar(stat = "identity") +
                geom_text(aes(x = domain,
                              y = count,
                              ymax = count,
                              label = paste(count, "(x", norm, ")"),
                              size = 3,
                              hjust= .5,
                              color = "white")) +
                labs(x = "Number of SNPs (nb SNPs/nb domains)",
                     y = "Pfam subsets")
```

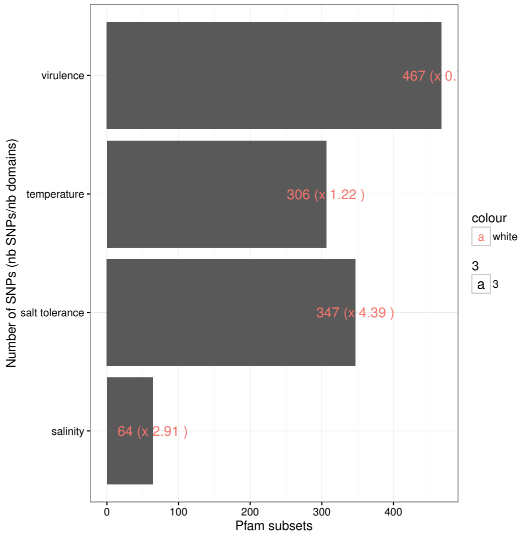

How many SNPs can be found outside of each domain? The outside SNPs can be upstream or downstream the aligned contig over the reference. The SNP position outside the domains is dependent on the Reference contig length, which was selected through alignment.

```
before <- c(155, 160, 11, 126)
after <- c(201, 89, 4, 163)
dat <- data.frame(domain, before, after)
dat <- gather(dat, "location", "count", 2:3)
ggplot(dat,
       aes(x = domain,
           y = count,
           fill = location)) +
    geom_bar(stat = "identity",
             position = "dodge") +
        theme_bw() +
            coord_flip() +
                geom_text(aes(x = domain,
                              y = count,
                              ymax = count,
                              label = count,
                              size = 3,
                              hjust = 2),
                          position = position_dodge(width = 1)) +
                labs(x = "Number of SNPs",
                     y = "Pfam subsets")
```

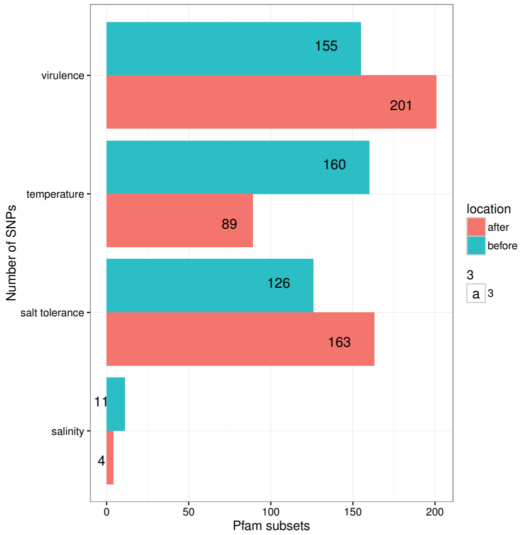

How many SNPs can be found inside and outside protein domains within each QPX strain?

```
before <- c(178, 21, 74, 133)
after <- c(177, 15, 69, 189)
inside <- c(593, 73, 216, 448)
strain <- c("m98", "m992", "m1002", "m1433")
dat <- data.frame(strain, before, inside, after)
dat <- gather(dat, "region", "count", 2:4)
ggplot(dat,
       aes(x = strain,
           y = count,
           fill = region)) +
    geom_bar(stat = "identity") +
        geom_text(aes(x = strain,
                      y = count,
                      ymax = count,
                      label = count,
                      vjust = 1,
                      size = 2),
                  position = "stack") +
            theme_bw() +
        labs(x = "QPX libraries",
             y = "SNP counts")
```

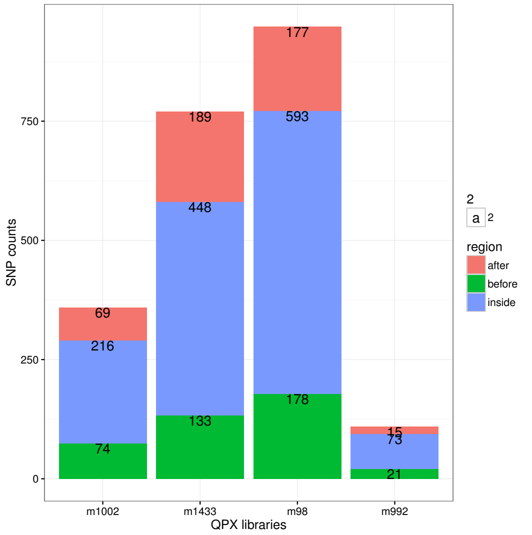

How many SNPs can be found inside and outside domains between virulence, temperature, salinity and within strain?

```r
dat <- read.xls("./data/hotspots/snps.all.pfam.xlsx", sheet = 1)
dat <- gather(dat, "region", "count", 3:5)
ggplot(dat,
       aes(x = lib,
           y = count,
           fill = region)) +
    geom_bar(stat = "identity") +
        geom_text(aes(x = lib,
                      y = count,
                      ymax = count,
                      label = count,
                      size = 1,
                      hjust = .5),
                  position = "stack") +
            facet_wrap( ~ pfam, ncol = 2) +
                theme_bw() +
                    scale_fill_hue(c = 40, l = 60) +
                labs(x = "QPX libraries",
                     y = "SNP counts")
```

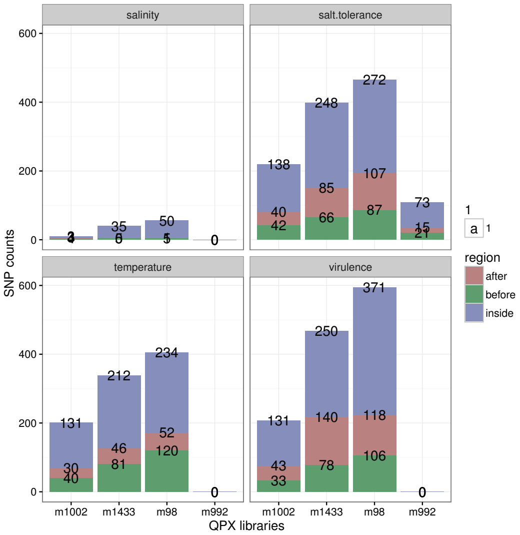

Preferential substitution inside/outside domains, per Pfam subset, and for each strain.

```r
dat <- read.xls("./data/hotspots/snps.all.pfam.xlsx", sheet = 2)
dat <- gather(dat, "mutation", "count", 3:14)
dat$mutation <- gsub(".", ">", dat$mutation, fixed = TRUE)
dat$pfam <- factor(dat$pfam, levels = c("virulence",
                                        "temperature",
                                        "salt.tolerance",
                                        "salinity"))
dat$lib <- factor(dat$lib, levels = c("m98",
                                      "m1002", "m1433", "m992"))
ggplot(dat,
       aes(x = mutation,
           y = count,
           fill = region)) +
    geom_bar(stat = "identity") +
            theme_bw() +
                facet_wrap(lib ~ pfam, ncol = 4) +
                    scale_fill_hue(c = 40,  l = 60) +
                labs(x = "Nucleotide substitution within called SNPs",
                     y = "SNP count")
```

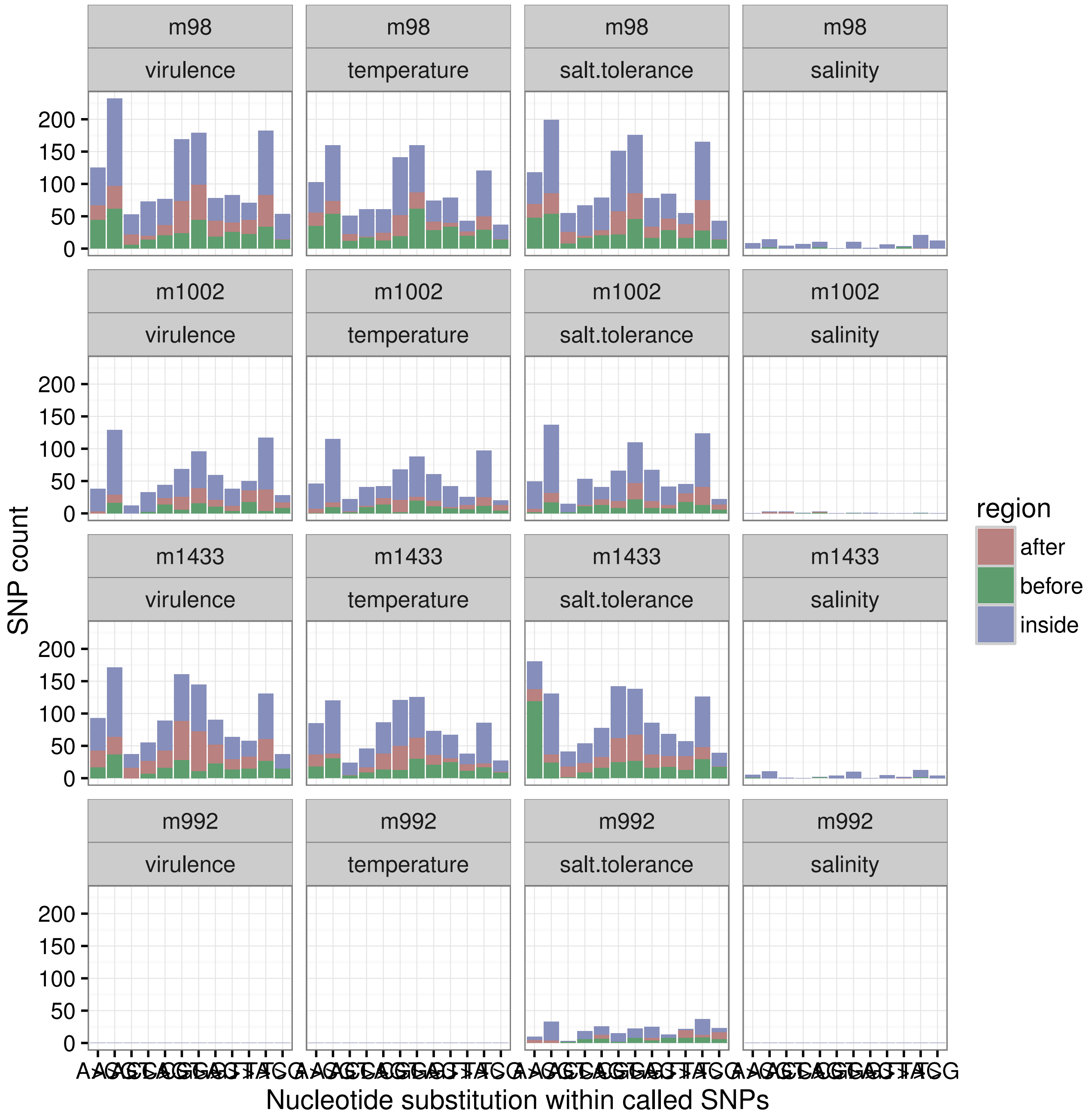

Frequency of SNPs inside Pfam domains for each strain, per 1 Kbp. Normalized by the total size of contigs for each strain.

```
dat <- read.xls("./data/hotspots/snps.all.pfam.xlsx", sheet = 1)
```

```r
inside <- c(593, 73, 216, 448)
sizen <- c(425098, 46409, 242136, 337206)
dat$inside <- with(dat, (inside/tsum)*1000)
ggplot(dat,
       aes(x = lib,
           y = inside)) +
    geom_bar(stat = "identity") +
        geom_text(aes(x = lib,
                      y = inside,
                      ymax = inside,
                      label = round(inside, digits = 2),
                      size = 1,
                      color = "white",
                      vjust = 1.5)) +
            facet_wrap( ~ pfam, ncol = 2) +
                theme_bw()

Warning:  Removed 3 rows containing missing values (position_stack).
Warning:  Removed 3 rows containing missing values (geom_text).
```

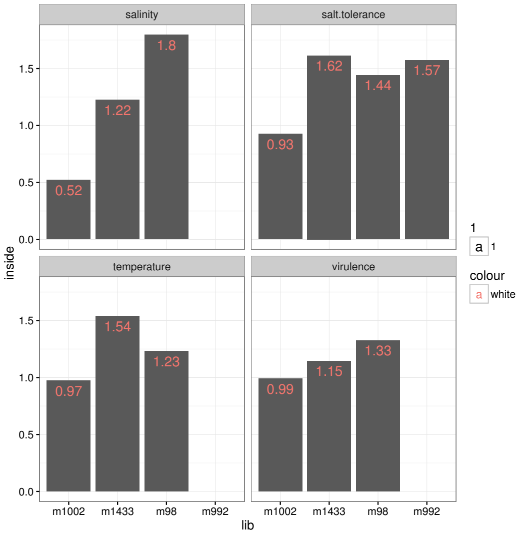

11 Machine learning on SNP hotspots of 4 QPX strains

Lets try a support vector machine classifier to differentiate between the QPX strains using quality data (above) of the variants. In progress ...

12 System Information

The version number of R and packages loaded for generating the vignette were:

```r
###save(list=ls(pattern=".*|.*"),file="PD.Rdata")
```

```r
sessionInfo()
```

```
R version 3.3.1 (2016-06-21)
Platform: x86_64-pc-linux-gnu (64-bit)
Running under: elementary OS Luna

locale:
 [1] LC_CTYPE=en_US.UTF-8       LC_NUMERIC=C
 [3] LC_TIME=en_US.UTF-8        LC_COLLATE=en_US.UTF-8
 [5] LC_MONETARY=en_US.UTF-8    LC_MESSAGES=en_US.UTF-8
 [7] LC_PAPER=en_US.UTF-8       LC_NAME=C
 [9] LC_ADDRESS=C               LC_TELEPHONE=C
[11] LC_MEASUREMENT=en_US.UTF-8 LC_IDENTIFICATION=C

attached base packages:
[1] grid      stats     graphics  grDevices utils     datasets
[7] methods   base

other attached packages:
 [1] tidyr_0.5.1         vegan_2.4-0         permute_0.9-0
 [4] dplyr_0.5.0         latticeExtra_0.6-28 RColorBrewer_1.1-2
 [7] lattice_0.20-33     gdata_2.17.0        knitr_1.13
[10] ggbiplot_0.55       scales_0.4.0        plyr_1.8.4
[13] ggplot2_2.1.0

loaded via a namespace (and not attached):
 [1] Rcpp_0.12.5      cluster_2.0.4    magrittr_1.5
 [4] MASS_7.3-45      munsell_0.4.3    colorspace_1.2-6
 [7] R6_2.1.2         stringr_1.0.0    highr_0.6
[10] tools_3.3.1      parallel_3.3.1   nlme_3.1-128
[13] gtable_0.2.0     mgcv_1.8-12      DBI_0.4-1
[16] gtools_3.5.0     lazyeval_0.2.0   digest_0.6.9
[19] assertthat_0.1   tibble_1.0       Matrix_1.2-6
[22] formatR_1.4      evaluate_0.9     labeling_0.3
[25] stringi_1.1.1    compiler_3.3.1   RevoUtils_10.0.1
```
